# Visualizing Material Processing via Photoexcitation-Controlled Organic-Phase Aggregation-Induced Emission

**DOI:** 10.34133/2021/9862093

**Published:** 2021-06-07

**Authors:** Jian Gu, Bingbing Yue, Glib V. Baryshnikov, Zhongyu Li, Man Zhang, Shen Shen, Hans Ågren, Liangliang Zhu

**Affiliations:** ^1^State Key Laboratory of Molecular Engineering of Polymers, Department of Macromolecular Science, Fudan University, Shanghai 200438, China; ^2^College of Science, University of Shanghai for Science and Technology, Shanghai 200093, China; ^3^Division of Theoretical Chemistry and Biology School of Biotechnology, KTH Royal Institute of Technology, SE-10691 Stockholm, Sweden; ^4^Department of Physics and Astronomy, Uppsala University, Box 516, SE-751 20 Uppsala, Sweden

## Abstract

Aggregation-induced emission (AIE) has been much employed for visualizing material aggregation and self-assembly. However, water is generally required for the preparation of the AIE aggregates, the operation of which limits numerous material processing behaviors. Employing hexathiobenzene-based small molecules, monopolymers, and block copolymers as different material prototypes, we herein achieve AIE in pure organic phases by applying a nonequilibrium strategy, photoexcitation-controlled aggregation. This strategy enabled a dynamic change of molecular conformation rather than chemical structure upon irradiation, leading to a continuous aggregation-dependent luminescent enhancement (up to ~200-fold increase of the luminescent quantum yield) in organic solvents. Accompanied by the materialization of the nonequilibrium strategy, photoconvertible self-assemblies with a steady-state characteristic can be achieved upon organic solvent processing. The visual monitoring with the luminescence change covered the whole solution-to-film transition, as well as the in situ photoprocessing of the solid-state materials.

## 1. Introduction

Although organic luminogens tend to be monomeric in organic solvents with an invariant emission, they usually show distinct emission behavior in aggregated states [1–3]. Accordingly, a change of the luminescence properties upon aggregation can be used for visualizing material aggregation and self-assembly with different forms. Aggregation-induced emission (AIE) [4–11], referring to some type of organic luminophores that can reveal bright luminescence in aggregated or solid states by the suppression of the nonradiative decay, plays an important role in many fields of application, like displays and lighting [4–11], in information technology [12–15], and molecular probing [16–18]. However, water is largely required for the preparation of AIE aggregates (*e.g.*, adding water into THF for preparing most of AIE aggregates [4–11, 19–21] or conversely adding THF into water for aggregating some water-soluble AIEgens [22, 23]). This operation brings considerable limitation for numerous material processes, especially for those polymer materials that will perform sensitive nanoscale features by different solution processing. Therefore, we plan to develop AIE in pure organic phases (no phase transition) to break through the restriction of solvent type in visualizing the material processing, the task of which remains challenging but desired.

Recently, AIE materials that rely on specific chemical reactions have emerged [24–26], which make it possible to address the above hypothesis. Nevertheless, these cases have still been developed basically in the aqueous phase for biological usage [24–28]. The alternative way of producing an AIE effect by light can be more advantageous, because light stimulus is typically precise and rapid, and can provide contactless spatial and temporal control [29–33]. However, the difficulty to produce AIE in pure organic solvents by chemical or photochemical approaches could be the control of solubility difference between the reactant and the product. Instead, a photoexcitation-based physical process [34, 35], which can sufficiently utilize photons to facilitate the entire molecular motion, could be a suitable approach to change the molecular solubility before and after irradiation. Thus far, several nonequilibrium systems based on the photoexcitation principle have been developed by overcoming the ultrafast relaxation and dissipation of the excitation state energy [36–41]. Inspired by these nonequilibrium systems, we aim here to employ such a unique photophysical strategy into AIE-active luminescent materials to attend the aforementioned hypothesis.

**Figure 1:**
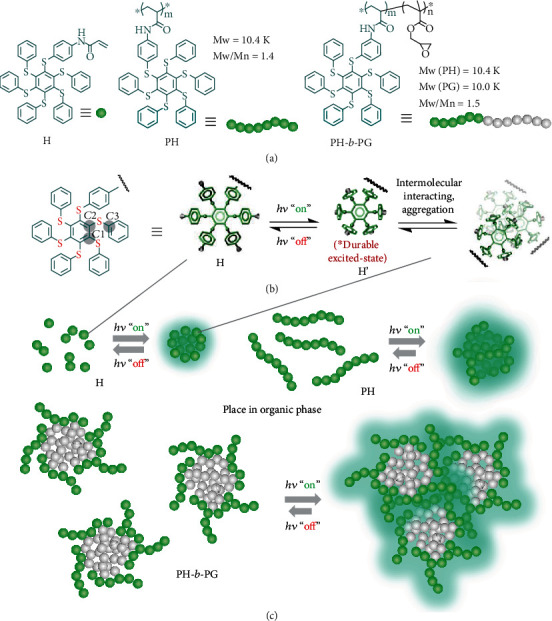
Outline for the photoexcitation-controlled organic-phase AIE: (a) chemical structures of monomer H, monopolymer PH, and block copolymer PH-*b*-PG based on hexathiobenzene; (b) the proposed conformational change upon photoexcitation of the hexathiobenzene-based skeleton. The related atoms were defined alongside the structure for the dihedral torsion analysis; (c) the illustration of photocontrolled organic-phase AIE accompanied by the molecular aggregation or morphological change of H, PH, and PH-*b*-PG.

In this work, hexathiobenzene-based small molecule, monopolymer, and block copolymer (H, PH, and PH-*b*-PG, see chemical structure in Figure 1, as well as the synthetic details in SI) were used for the study of photoexcitation-controlled organic-phase AIE. We take account of the fact that persulfurated aromatic molecules can serve as metal-free room-temperature phosphorescence emitters, for which an intersystem crossing process is environmentally adjustable. Due to this circumstance, persulfurated arenes can demonstrate unique aggregation-induced phosphorescence (AIP, an important class of AIE phenomena) [42, 43]. We fully utilized the asterisk-shaped molecular design of hexathiobenzene, which can show a significant conformational difference of molecular geometry between the ground and excited states. In addition, the durable excited-state of this luminophore guarantees a sufficient time lapse for molecular aggregation against excited-electron relaxation [44]. These factors can make it possible to achieve a photocontrollable wide-range organic-phase AIE. On the other hand, because the direct self-assembly (DSA) and the microphase-segregated feature of a block copolymer (BCP) is sensitive to the structure and solvent condition [45–47], we mainly focus on the BCP prototype before and after irradiation to highlight a visual monitoring of material self-assembly.

## 2. Results

### 2.1. AIE Behavior in Pure Organic Phases

**Figure 2:**
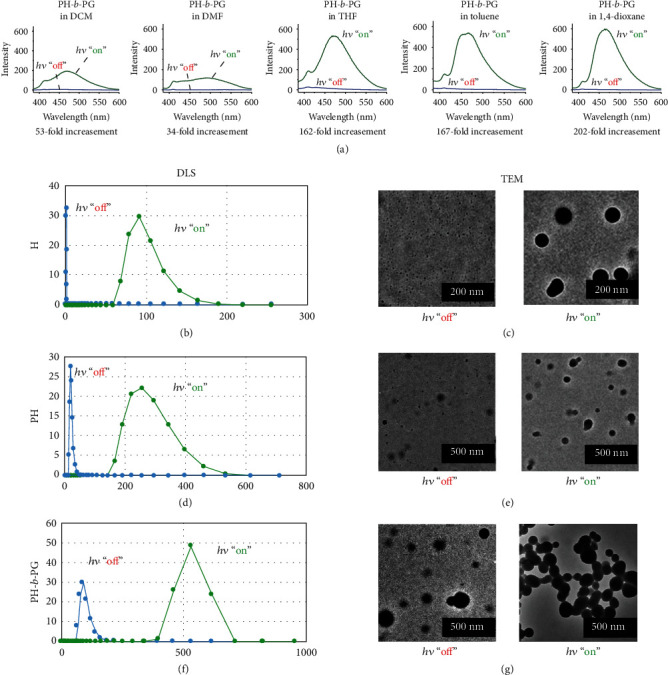
AIE behavior in pure organic phases: (a) emission spectra of PH-*b*-PG placed in different organic solvents before and after irradiation for 90 s. Spectra were collected upon 10 *μ*M of the hexathiobenzene unit at 298 K (*λ*_ex_ = 365 nm, measured in the same parameters). DLS results before and after irradiation for 90 s of (b) H, (d) PH, and (f) PH-*b*-PG, taken upon 10 *μ*M of the hexathiobenzene unit in 1,4-dioxane at 298 K. TEM image of a sample of (c) H, (e) PH, and (g) PH-*b*-PG, prepared from a corresponding 1,4-dioxane solution before and after irradiation for 90 s.

As the hexathiobenzene-based skeleton contributed largely to the optical properties of these materials (evidenced by the similar band wavelength location in absorptional spectra among H, PH, and PH-*b*-PG, see Fig. S3), we firstly explore the photoluminescent property in different solvents. When PH-*b*-PG is placed in a variety of organic solvents, it exhibits a typical significant enhancement of the emission signal upon continuous UV irradiation (Figure 2(a)), accompanied by an increase of the luminescent quantum yield up to ~200-fold. This indicates a possible photocontrolled AIE behavior in the organic phases. Due to the solvent difference, the degree of the light-controlled aggregation as well as the solubility of the aggregates will differ, thus leading to different luminescence increments of the AIEgen. As it possesses the highest emission intensity in the 1,4-dioxane solvent after UV irradiation, we focused on this organic solvent for the further studies.

To access the possible AIE behavior with a photoexcitation principle, a study of the molecular structure of the compounds was initially performed. No changes in the results of the fundamental characterization were observed before and after irradiation (see the NMR and FT-IR spectra in Figs. S4–S9), indicating that the molecular structure of these species remains invariant and that the possibility of a photochemical reaction process is eliminated. However, the molecular aggregation behavior upon irradiation can be monitored by DLS and TEM studies. They show that the relatively well-dispersed nanometer-sized parts (less than 20 nm in diameter) of H and PH form larger nanometer-sized aggregates (more than 90 nm in diameter) after irradiation (Figures 2(b)–2(e)). On the other hand, PH-*b*-PG can initially form micelles [48–50] with an average diameter of 95 nm in 1,4-dioxane, and the micelles can be cross-linked upon UV irradiation (Figures 2(f) and 2(g)).

**Figure 3:**
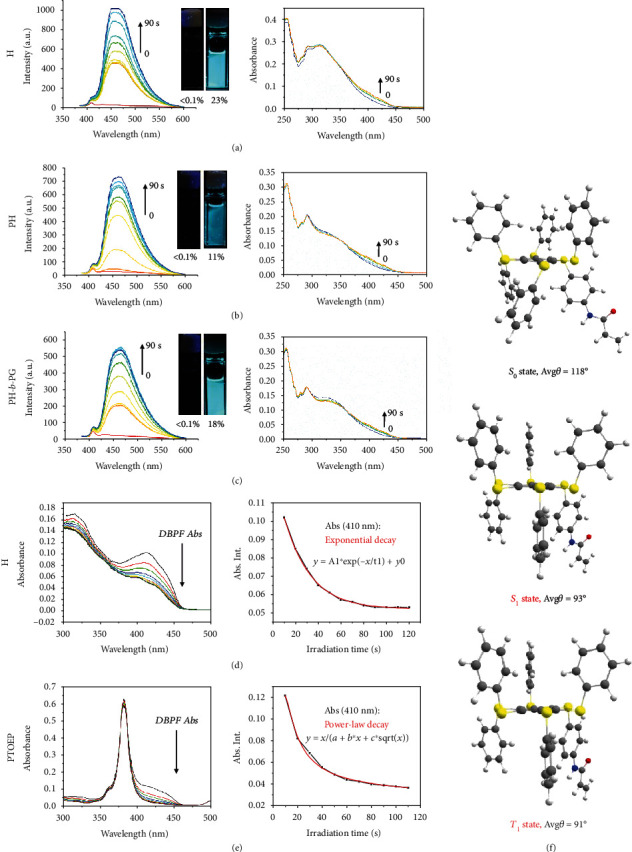
Mechanism study: continuous enhancement of emission and absorption spectra of (a) H, (b) PH, and (c) PH-*b*-PG in 1,4-dioxane with prolonged irradiation duration. The insert shows the corresponding photographs before and after irradiation under a UV lamp. The absorption and absorption intensity at 410 nm of DPBF in the presence of (d) H and (e) PTOEP versus the UV irradiation time for monitoring the singlet-oxygen formation and the triplet-state behavior. (f) Calculated geometry of the ground (*S*_0_ state) and excited states (*S*_1_ and *T*_1_ states) of H.

It is likely that such an aggregation tendency is responsible for the AIE, as the nearly quenched emission of the three compounds was found to appear immediately upon irradiation, and the emission signal (~470 nm) became stronger over time (Figures 3(a)–3(c)). This behavior is synchronously accompanied by a small change of the absorption band at >400 nm. Such a photocontrolled AIE effect can be well visualized (see the corresponding insert photographs in Figures 3(a)–3(c)). Interestingly, the emission of these solutions is self-recoverable after the removal of the irradiation (see the emission signal decreased quickly at 470 nm, Fig. S10). Such reversibility further indicates a nonequilibrium photoexcitation feature. Due to entanglement, the recovery of the polymers (PH and PH-*b*-PG) is slower than that of the monomer H.

### 2.2. Mechanism Study

The mechanism of photoexcitation-controlled aggregation of the hexathiobenzene-based monomer and polymers can be explored from two perspectives, namely, from the population of the long-lifetime excited states and from the molecular conformational change upon photoexcitation. The formation of singlet oxygen, indicated by the reduction in the absorption of a quencher (1.3-diphenylisobenzofuran, DPBF) [51, 52], reflects the triplet-state behavior of the hexathiobenzene skeleton of H (Figure 3(d)). However, as compared to the power-law trend in DPBF absorption decay in the presence of the classic porphyrin photosensitizer (platinum(II) octaethylporphine, PTOEP, see Figure 3(e)), the decay of DPBF absorption exhibits an exponential trend in the presence of H (Figure 3(d)). This indicates a continuous variation of the triplet-state behavior of hexathiobenzene [53] upon the photoexcitation-induced molecular aggregation process (see also a full comparison in Fig. S11). In addition, we also access the ground- and excited-state conformations of H by theoretical calculations. As shown in Figure 3(f), the average dihedral torsion *θ* (defined by those atoms labeled in Figure 1) of H in the excited-state conformation is much smaller than that in the ground-state conformation (118° in the *S*_0_ state *vs.* 93° in the *S*_1_ state and 91° in the *T*_1_ state, see also detailed geometric parameters in Table S1). This suggests that the phenyl substituents are positioned more perpendicularly to the plane of the inner benzene core upon photoexcitation, facilitating the molecular aggregation process.

**Figure 4:**
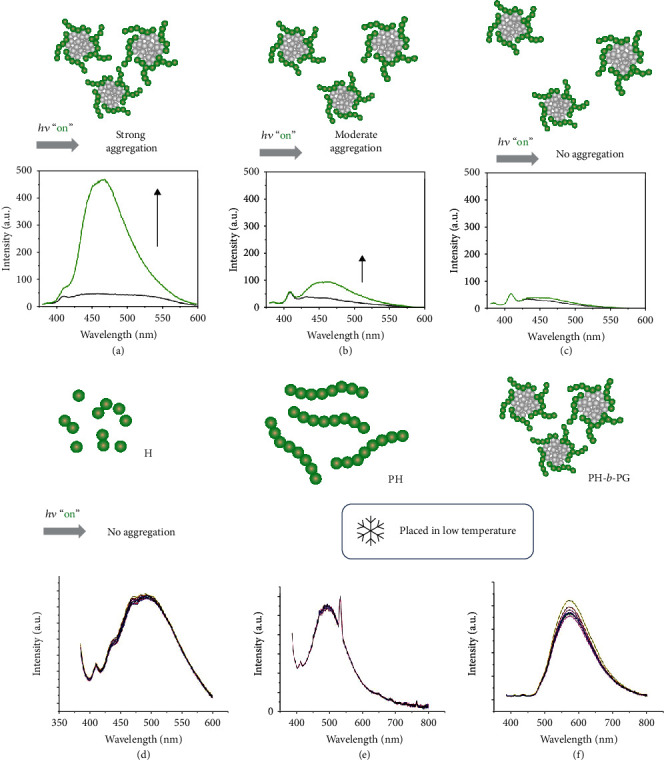
Molecular motion exploration: emission enhancements of PH-*b*-PG in 1,4-dioxane before and after irradiation for 10 s with the concentration of hexathiobenzene unit at (a) 0.1 mM, (b) 0.01 mM, and (c) 0.001 mM. Emission spectra of (d) H, (e) PH, and (f) PH-*b*-PG in 1,4-dioxane with continuous UV irradiation at low temperature (less than 150 K).

The concentration-dependent irradiation experiment of PH-*b*-PG further verified the above mechanism. Because high concentration is more beneficial to intermolecular interaction and collision than low concentration, it is conducive to the photoexcitation-controlled aggregation and the luminescence enhancement (Figures 4(a)–4(c)). On the other hand, when the temperature was reduced, the emission enhancement of these compounds became less sensitive upon irradiation (Figures 4(d)–4(f)), indicating that the molecular motion induced by photoexcitation can be inhibited at low temperature (<150 K).

### 2.3. Visualizing Organic Solution Processing

**Figure 5:**
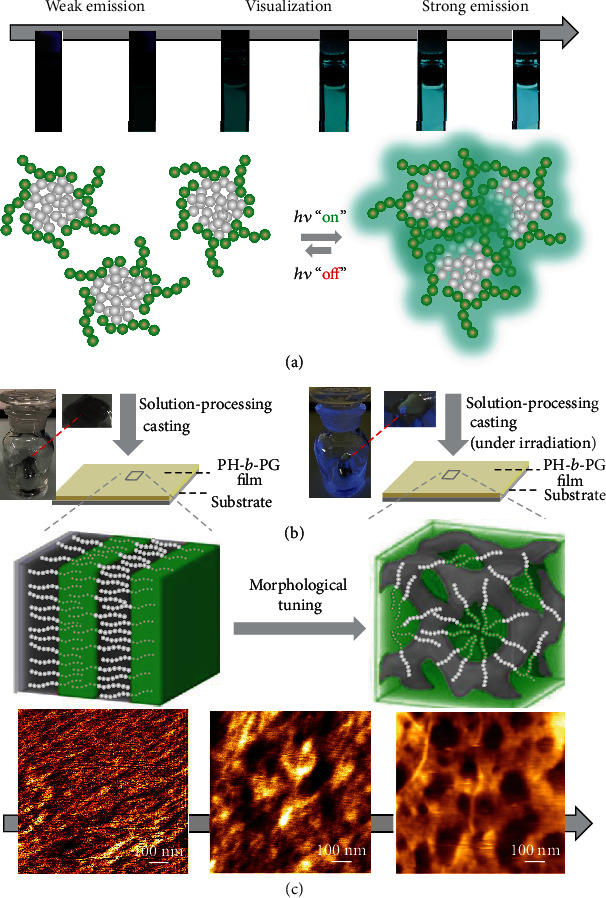
Visualizing organic solution processing: (a) illustration of the employment of photoexcitation-controlled AIE behavior to visualize the DSA of PH-*b*-PG with different morphologies, accompanied by a continuous luminescent enhancement upon irradiation; (b) the films prepared by casting of PH-*b*-PG solution before and after irradiation onto mica substrates and the photograph of the films placed in a closed jar with the 1,4-dioxane vapor for annealing; (c) corresponding AFM height images of the PH-*b*-PG films showing a photoconvertible microphase-segregated DSA from lamellar to a cross-linked feature upon irradiation.

As BCPs can exhibit unique DSA upon solution processing [45–47], we utilize the aforementioned photoexcitation-controlled AIE behavior to visualize the DSA of PH-*b*-PG (Figure 5(a)). Films prepared by casting of PH-*b*-PG solution before and after irradiation onto mica substrates were placed in a closed jar for solvent annealing (Figure 5(b)). Different from the lack of nanoscale feature of H and PH (Fig. S12), a typical lamellar microphase-segregated feature could be observed from the DSA of PH-*b*-PG without irradiation, largely depending on the close volume fraction of the two blocks (see AFM image in Figure 5(c)) [54]. When DSA took place from the solution of PH-*b*-PG upon irradiation, the photocontrolled aggregation of the hexathiobenzene-based block will drive both of the two blocks as a whole for realignment, causing a photoconvertible microphase-segregated DSA from a lamellar to a cross-linked feature (Figure 5(c)). Both the DSAs before and after irradiation are amorphous at the molecular scale, evidenced by the broad XRD signals (Fig. S13).

### 2.4. Visualizing In Situ Photoexcitation Control

**Figure 6:**
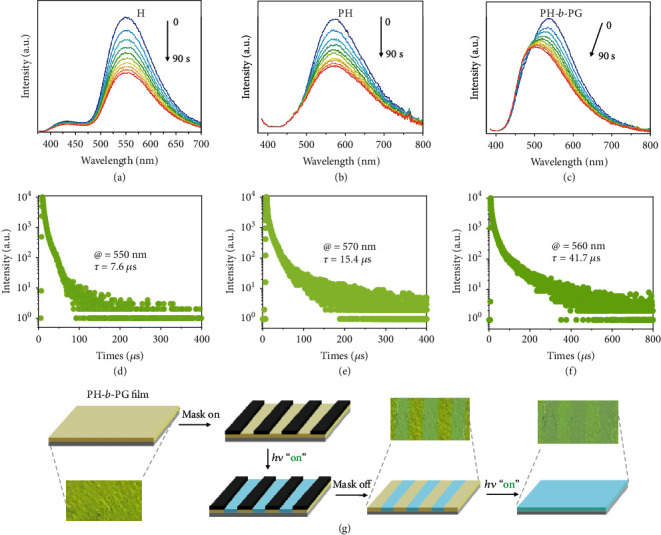
Visualizing in situ photoexcitation control: continuous change of emission of (a) H, (b) PH, and (c) PH-*b*-PG in the film state with prolonged irradiation duration. Corresponding photoluminescent lifetime spectra of (d) H, (e) PH, and (f) PH-*b*-PG in the film state. (g) The visualized photopatterning experiment performed on a PH-*b*-PG film with regionally selective irradiation.

Upon on the organic solution processing with the visualization of the organic-phase AIE, we demonstrate more practical applications of these materials by photoexcitation-controlled molecular motion conducted directly in the film state under in situ irradiation. As the hexathiobenzene itself is a typical AIP luminophore, the film state of which, without additional doping matrix, initially exerts a strong emission in these compounds (Figures 6(a)–6(c)). This is due to the fact that the molecules are already in a condensed state and thus inhibit the nonradiative decay. In these cases, a microsecond scale lifetime that signified an ambiguous phosphorescence emission can be well measured (Figures 6(d)–5(f)). After irradiation, the photoexcitation-controlled molecular motion caused the aggregation change, leading to molecules that turned into another condensed state with the emission reduced a little (Figures 6(a)–6(c)).

Different from the solution state, the self-recoverability of the film state of these materials and their emission signals are passivated (Fig. S14) because of the kinetic trapping effect in the solid state [55, 56]. Compared with the monomer H, some weight loss (*ca.* 5%) of PH-*b*-PG before the thermal decomposition temperature is observed (<200°C, Figure S15), which is usually related to the bond breaking of the polymer chain. However, we can find that such a weight loss of PH-*b*-PG was postponed after photoirradiation (Figure S15), showing an enhanced thermal stability upon the change of aggregation. This can also explain the irreversibility of the photoexcitation-controlled molecular motion in the solid state, a behavior of which is beneficial for a steady-state application.

Motivated by the observations reviewed above, we perform an application showcase of PH-*b*-PG with photoirradiation. As hexathiobenzene is a luminophore with charge-transfer (CT) nature [42, 43], it is also sensitive to the polarity of the microenvironment [57]. The nonpolar block PG can affect the microenvironment of the hexathiobenzene luminophore upon the photoconvertible microphase-segregated self-assembly (referenced by the depiction in Figure 5(c)), leading to a further emission blue-shift in PH-*b*-PG (Figure 6(c)) relative to the unshifted emission in H and PH (Figures 6(a) and 6(b)). In solution, we did not observe such an emission shift because the nonpolar block PG is relatively free. By employing this photocontrolling property of PH-*b*-PG, a visualized photopatterning experiment, without additional doping matrix, can be performed. As shown in Figure 6(g), when the PH-*b*-PG film was alternately covered by masks, the regionally selective irradiation of the film gives a desired pattern showing emission stripes (see a cyan luminescence color relative to the yellow one alternate). The emission change induced by irradiation still continues after the masks are removed. This performance is advantageous for in situ photoexcitation-based control, indicating an application potential as functional materials with information processing [58, 59].

## 3. Discussion

This study presents a unique strategy for achieving organic-phase AIE. It is based on a nonequilibrium process involving photoexcitation-induced molecular aggregation. The strategy is illustrated by an asterisk-shaped molecular design of the hexathiobenzene luminophore, which exerts a relatively large molecular conformational difference between its ground and excited states. On the other hand, the long lifetime of the excited-state guarantees a sufficient time lapse for molecular aggregation against the electron relaxation. As exemplified by the photoconvertible self-assembly accompanied by the organic-phase AIE, a visual monitoring of the solution processing of the materials with different morphologies could be achieved. Totally different from the previously reported examples of photoexcitation strategy with self-relaxation, a steady-state design conception was introduced here, moving forward the materialization of the photoexcitation-based physical strategy for material processing.

## Data Availability

All data needed in the paper are present in the paper and in the Supplementary section. Additional data which are related to this paper may be requested from the authors.

## Supplementary Materials

Supplementary Materials Figure S1: synthetic route for H, PH, and PH-*b*-PG. Figure S2: GPC spectra of PH and PH-*b*-PG. Figure S3: absorption spectra of H, PH, and PH-*b*-PG. Spectra were collected upon 10 *μ*M of the hexathiobenzene unit in 1,4-dioxane at 298 K. Figure S4: 1HNMR spectra of H in CDCl3 upon a continuous UV irradiation. Figure S5: FTIR spectra of H before and after UV irradiation for 3 min. Figure S6: 1HNMR spectra of PH in CDCl3 upon a continuous UV irradiation. Figure S7: FTIR spectra of PH before and after UV irradiation for 3 min. Figure S8: 1HNMR spectra of PH-*b*-PG in CDCl3 upon a continuous UV irradiation. Figure S9: FTIR spectra of PH-b-PG before and after UV irradiation for 3 min. Figure S10: the emission changes of H, PH, and PH-*b*-PG in 1,4-dioxane after irradiation for 90 s, followed by the relaxation without irradiation for the same period. Table S1: selected torsion angles (*θ*, deg.) for the different conformers of H. Figure S11: UV-vis absorption spectra after irradiation at 365 nm. Figure S12: AFM height images of (a) H and (b) PH. Figure S13: the XRD spectra of PH-*b*-PG before and after UV irradiation for 10 min. Figure S14: the emission changes of H, PH, and PH-*b*-PG in the film state after irradiation for 90 s, followed by the relaxation without irradiation for the same period. Figure S15: TGA of (a) H and (b) PH-*b*-PG before and after irradiation and (c) a partially magnified spectrum of (b).
