# Supplementary material for: Visualizing Material Processing via Photoexcitation-Controlled Organic-Phase Aggregation-Induced Emission
Source: Research (Wash D C). 2021 Jun 7;2021:9862093. doi: 10.34133/2021/9862093 (PMC8208088; doi:10.34133/2021/9862093)
Supplement: Supplementary Materials — Figure S1: synthetic route for H, PH, and PH-b-PG. Figure S2: GPC spectra of PH and PH-b-PG. Figure S3: absorption spectra of H, PH, and PH-b-PG. Spectra were collected upon 10 μM of the hexathiobenzene unit in 1,4-dioxane at 298 K. Figure S4: 1HNMR spectra of H in CDCl3 upon a continuous UV irradiation. Figure S5: FTIR spectra of H before and after UV irradiation for 3 min. Figure S6: 1HNMR spectra of PH in CDCl3 upon a continuous UV irradiation. Figure S7: FTIR spectra of PH before and after UV irradiation for 3 min. Figure S8: 1HNMR spectra of PH-b-PG in CDCl3 upon a continuous UV irradiation. Figure S9: FTIR spectra of PH-b-PG before and after UV irradiation for 3 min. Figure S10: the emission changes of H, PH, and PH-b-PG in 1,4-dioxane after irradiation for 90 s, followed by the relaxation without irradiation for the same period. Table S1: selected torsion angles (θ, deg.) for the different conformers of H. Figure S11: UV-vis absorption spectra after irradiation at 365 nm. Figure S12: AFM height images of (a) H and (b) PH. Figure S13: the XRD spectra of PH-b-PG before and after UV irradiation for 10 min. Figure S14: the emission changes of H, PH, and PH-b-PG in the film state after irradiation for 90 s, followed by the relaxation without irradiation for the same period. Figure S15: TGA of (a) H and (b) PH-b-PG before and after irradiation and (c) a partially magnified spectrum of (b). [file 9862093.f1.doc]

Supplementary Information for:

**Visualizing Material Processing via Photoexcitation-Controlled Organic-Phase Aggregation-Induced Emission**

Jian Gu,^1^ Bingbing Yue, *^2^ Glib V. Baryshnikov,^3^ Zhongyu Li,^1^ Man Zhang,^1^ Shen Shen,^1^ Hans Ågren,^4^ and Liangliang Zhu*^1^

^1^ State Key Laboratory of Molecular Engineering of Polymers, Department of Macromolecular Science, Fudan University, Shanghai 200438, China.

^2^ College of Science, University of Shanghai for Science and Technology, Shanghai 200093, China

^3^ Division of Theoretical Chemistry and Biology School of Biotechnology, KTH Royal Institute of Technology, SE-10691 Stockholm, Sweden

^4^ Department of Physics and Astronomy, Uppsala University, Box 516, SE-751 20 Uppsala, Sweden

^1*^ Correspondence to: zhuliangliang@fudan.edu.cn (L. Z.); yuebingbing@usst.edu.cn (B. Y.)

General Information

All reagents were purchased from Aldrich and used without additional purification except glycidyl methacrylate which was treated with aluminum oxide before use. ^1^H NMR and ^13^C NMR spectra were measured on a Bruker 400L spectrometer. High‐resolution mass spectrometry (HRMS) data was measured by Matrix assisted laser desorption ionization-time of flight/time of flight mass spectrometer (5800). Molecular weight of the synthesized polymer was measured with an Agilent 1269 gel permeation chromatography (GPC) equipped with a UV detector and calibrated with polystyrene standard samples. The UV-vis absorption spectra were recorded on a Shimadzu 1800 spectrophotometer. The emission spectra were recorded on Shimadzu RF-5301, and the lifetime spectra were recorded on FLS 920 (Edinburgh Instruments). The photoirradiation experiments for the photoexcitation-induced molecular aggregation strategy were carried out using a hand-held UV lamp (with the irradiation wavelength of 365 nm in a sealed 10 mm quartz cell; the power of the UV lamp is 5W; the distance between the sample and the lamp is ~10 cm; the light receiving area of the sample is estimated to be about 0.5 cm^2^). The fluorescence quantum yields of solution were measured on QM40 with an integrating sphere (φ 150 mm) from Photo Technology International, Inc. (PTI, USA). The topography and separated phase of the films were analyzed with an AFM (Bruker Multimode 8) in tapping mode using a silicon tip with aluminum reflex coating (Bruker RTEASPA300). Transmission electron microscopy (TEM) was performed on a Jeol JEM 2100 with an accelerating voltage of 200 kV. Fourier transform infrared (FTIR) spectroscopy was carried out with a Thermofisher Nicolet 6700 spectrometer using KBr pellets as the sample matrix in the wavenumber range of 400-4000 cm^-1^. Dynamic light scattering (DLS) experiments were carried out with Nano-Zeta Potential Analyzer ZS-90. TG analyses were performed on a Metller TG instrument under a dry nitrogen atmosphere at a heating rate of 20 °C min^−1^. DSC analyses were performed with a TAQ2000 instrument under nitrogen atmosphere at a heating rate of 20 °C min^−1^.

Figure S1. Synthetic route for **H**, **PH**, and **PH-*b*-PG**.

**Synthesis of compound 1:** Perfluorobenzene (186 mg, 1 mmol), sodium benzenethiolate (520 mg, 4 mmol) were added into a round bottom flask capped with a septum under an argon atmosphere. DMF (20 mL) was injected via a syringe and the mixture was stirred at room temperature for 48 h. Water (20 mL) was poured into the flask while stirring, and a yellow precipitate was obtained. After collecting the solid by filtration, it was rinsed with ethanol and water. The as prepared powder was purified in silica gel column with petroleum ether (PE) and dichloromethane (DCM) (10:1) and then dried under high vacuum. ^1^H NMR (400 MHz, dmso-*d*) δ 7.19-7.31 (m, 20 H). ^13^C NMR (101 MHz, chloroform-*d*) δ 160.59, 158.10, 134.71, 129.48, 129.11, 128.88 (d), 127.05. MS: MALDI-TOF MS, m/z: calcd for C_30_H_20_F_2_S_4_, 546.04; found m/z, 546.11.

**Synthesis of compound 2:** Compound **1** (3276 mg, 6 mmol), sodium benzenethiolate (2376 mg, 18 mmol)，4-Acetamidothiophenol (2004 mg, 12 mmol), K_2_CO_3_ (1656mg, 12mmol) were added into a round bottom flask capped with a septum under an argon atmosphere. DMF (60 mL) was injected via a syringe and the mixture was stirred at 60 ^o^C for 24 h. Upon completion, saturated brine was added, and extracted with EtOAc. The volatiles were removed under vacuum to afford the crude product. The crude product was purified by column chromatography on silica gel with PE and EtOAc (2:1) and then dried under high vacuum. ^1^H NMR (400 MHz, Chloroform-*d*) δ 7.34 (d, J = 8.7 Hz, 2H), 7.20 – 7.10 (m, 15H), 7.01 – 6.89 (m, 12H), 2.19 (s, 3H). ^13^C NMR (101 MHz, Chloroform-*d*) δ 168.25，148.60, 148.14, 147.92，147.66, 137.67, 136.57，132.55, 129.62, 128.98, 128.20, 128.15, 128.10, 126.22, 126.18, 120.11, 24.65. MS: MALDI-TOF MS, m/z: calcd for C_44_H_33_NOS_6_, 783.09; found m/z, 782.97.

**Synthesis of compound 3:** Compound **2** (784 mg, 1 mmol) was added into a three neck round bottom flask capped with a septum under an argon atmosphere. 1,4-dioxane (5 mL) and HCl (12 M, 2 mL) were injected via syringes and the mixture was stirred at 110 ^o^C for 24 h. Upon completion, K_2_CO_3_ and saturated brine were added, and extracted with EtOAc. The volatiles were removed under vacuum to afford the crude product. The crude product was purified by column chromatography on silica gel with PE and DCM (2:1) and then dried under high vacuum. ^1^H NMR (400 MHz, Chloroform-*d*) δ 7.21 – 7.08 (m, 15H), 7.01 – 6.87 (m, 12H), 6.50 (d, J = 8.6 Hz, 2H), 3.67 (s, 2H). ^13^C NMR (101 MHz, Chloroform-*d*) δ 150.52, 147.83, 147.32, 146.97, 145.18, 137.94, 137.83, 137.82, 132.04, 128.97, 128.89, 128.85, 128.10, 128.01, 126.01, 125.99, 125.62, 115.78. MS: MALDI-TOF MS, m/z: calcd for C_42_H_31_NS_6_, 741.08; found m/z, 740.96.

**Synthesis of H:** Compound **3** (370 mg, 0.5 mmol) was added into a round bottom flask capped with a septum under an argon atmosphere. DCM (5 mL) and triethylamine (0.5 mL) were injected via syringes，acryloyl chloride (0.5 mL) was added dropwise under 0 ^o^C and the mixture was stirred at room temperature for 16 h. Upon completion, the volatiles were removed under vacuum to afford the crude product. The crude product was purified by column chromatography on silica gel with PE and DCM (1:1) and then dried under high vacuum. ^1^H NMR (400 MHz, Chloroform-*d*) δ 7.51 (d, J = 40.9 Hz, 2H), 7.15 (dd, J = 15.5, 6.7 Hz, 15H), 6.97 (d, J = 7.1 Hz, 12H), 6.44 (d, J = 16.8 Hz, 1H), 6.24 (dd, J = 16.8, 10.2 Hz, 1H), 5.76 (d, J = 10.3 Hz, 1H). ^13^C NMR (101 MHz, Chloroform-*d*) δ 148.51, 148.18, 147.97, 147.68, 137.67, 136.46, 132.92, 131.05, 129.60, 129.00, 128.22, 128.17, 128.11, 126.25, 126.20, 120.30. MS: MALDI-TOF MS, m/z: calcd for C_45_H_34_NOS_6_, 795.09; found m/z, 795.01.

Synthesis of PH: To a 25 mL Schlenk flask, equipped with a Teflon-coated stir bar, was added H (160 mg, 0.2 mmol), the transfer agent (4-cyano-4-(((ethylthio)carbonothioyl)thio)pentanoic acid, 5.4 mg, 0.02 mmol), AIBN (0.64 mg, 0.004 mmol), and distilled dioxane (2 mL). The flask was capped with a rubber septum and the mixture was stirred until all the stuff dissolved, resulting in a clear yellow solution that was degassed by three cycles of freeze/pump/thaw. After the final thaw cycle, the flask was backfilled with Ar and the flask was sealed and immersed in a 75 °C oil bath for 24 h. Then the flask was immersed in liquid nitrogen to quench the polymerization. The frozen mixture was allowed to warm to room temperature and the crude polymer was then precipitated into a 1:1 mixture of icy water and methanol from minimal dioxane three times. The precipitates were filtered out and dried in vacuum overnight to yield 120 mg of yellow polymer compound. Mn = 7200 Da, Mw = 10400 Da, Mw/Mn = 1.4. The polymerization degree of H is ∼13. ^1^H NMR (400 MHz, CDCl_3_, 298K): δ = 7.17−7.11 (br), 6.96−6.94 (br).

Synthesis of PH-b-PG: To a 25 mL Schlenk flask, equipped with a Teflon-coated stir bar, was added PH (80mg), glycidyl methacrylate (GMA) (100 mg, 0.7 mmol), AIBN (1.6 mg, 0.01 mmol), and distilled dioxane (2 mL). The flask was capped with a rubber septum and the mixture was stirred until all the stuff dissolved, resulting in a clear yellow solution that was degassed by three cycles of freeze/pump/thaw. After the final thaw cycle, the flask was backfilled with Ar and the flask was sealed and immersed in a 75 °C oil bath for 24 h. Then, the flask was immersed in liquid nitrogen to quench the polymerization. The frozen mixture was allowed to warm to room temperature and the crude polymer was then precipitated into methanol from minimal dichloromethane three times. The precipitates were filtered out and dried in vacuum overnight to yield 100 mg of yellow polymer compound. Mn = 13 700 Da, Mw =20 400 Da, Mw/Mn = 1.5. Mw (PH) = 10.4 K, Mw (PG) = 10.0 K. The polymerization degrees of H and G are ∼13 and ∼70, respectively. ^1^H NMR (400 MHz, CDCl_3_, 298 K): δ = 7.17−7.11 (br), 7.02−6.79 (br), 4.37−4.31 (br), 3.86−3.80 (br), 3.31−3.21 (br), 2.92−2.81 (br), 2.69−2.60 (br), 1.92−1.74 (br), 1.11−0.96 (br).


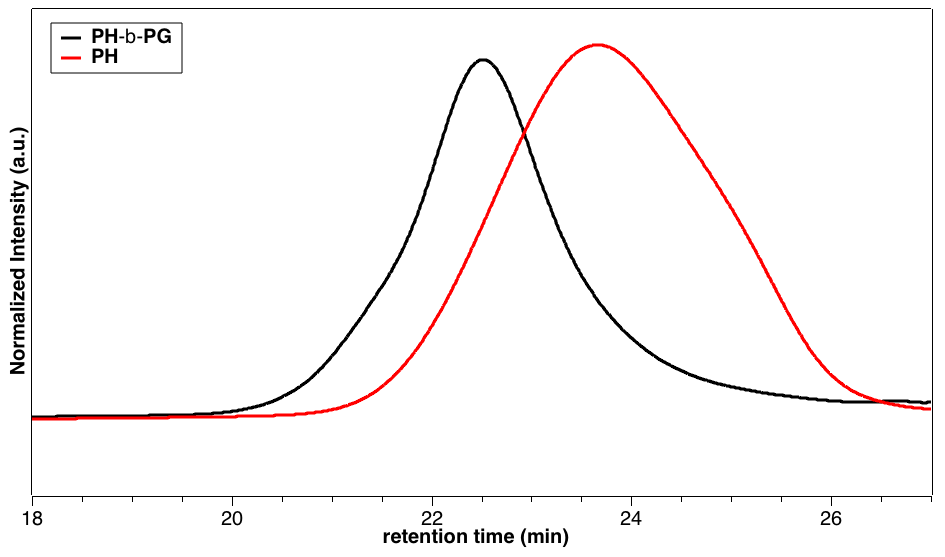


Figure S2. GPC spectra of **PH**, and **PH-*b*-PG**.

**Preparation of PH-*b*-PG Film**: 10 μL of 10 wt% **PH-*b*-PG** solution in toluene was coated on the silicon wafers which were precleaned by organic solvent. Subsequently, coated siliconwafers were placed on the holder with toluene vapor in room temperature for 16 hours.

Computational details: The structure of H was initially optimized by B3LYP/6-31G (d) ^[S1-S3]^ method in the ground singlet (S_0_) state following the [3, 3] conformation of phenyl substituents (6 phenyl groups are placed up and down relative to the central C_6_S_6_ core plane in alternation order). Starting from S_0_ optimized geometry, the structures of S_1_ and T_1_ states were optimized by TDDFT/B3LYP/6-31G(d) ^[S4]^ and UB3LYP/6-31G(d) methods, respectively. All calculations were performed by using Gaussian 16 (Revision A.03) software [https://gaussian.com/citation/].


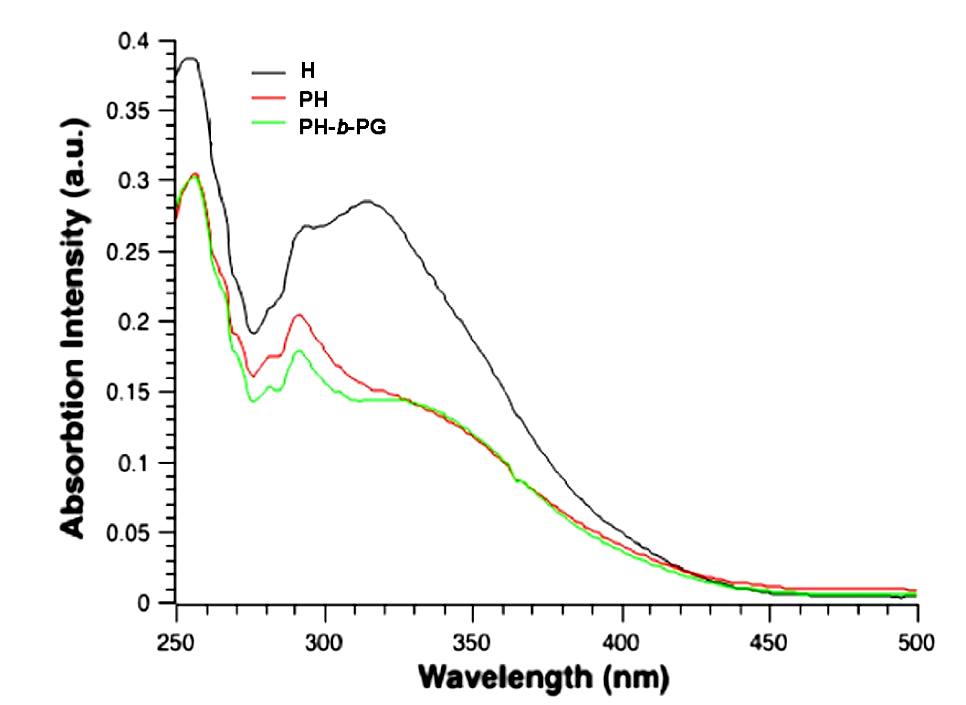


Figure S3. Absorption spectra of **H**, **PH**, and **PH-*b*-PG**. Spectra were collected upon 10 μM of the hexathiobenzene unit in 1, 4-dioxane at 298 K.


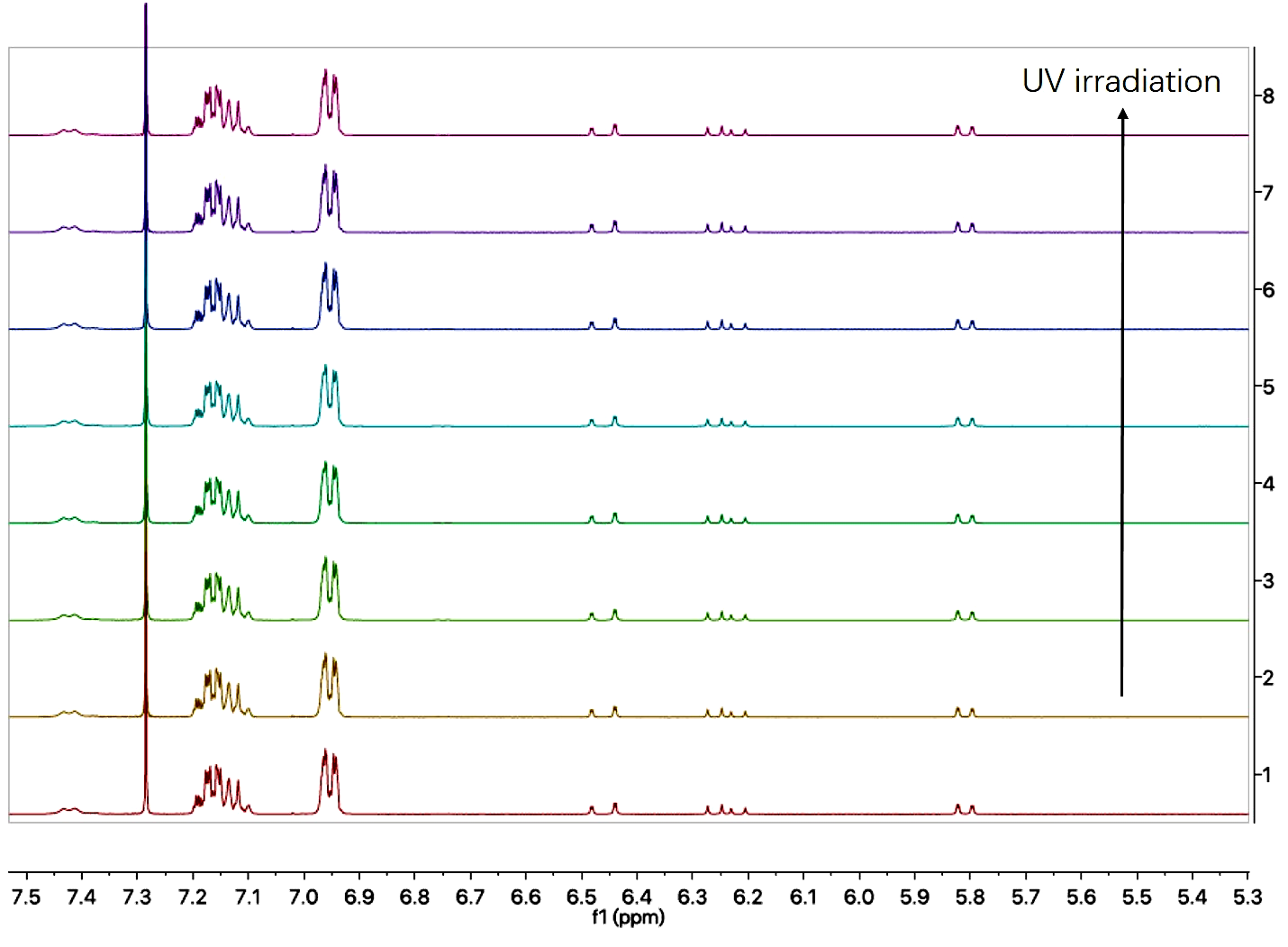


Figure S4. ^1^HNMR spectra of **H** in CDCl_3_ upon a continuous UV irradiation.


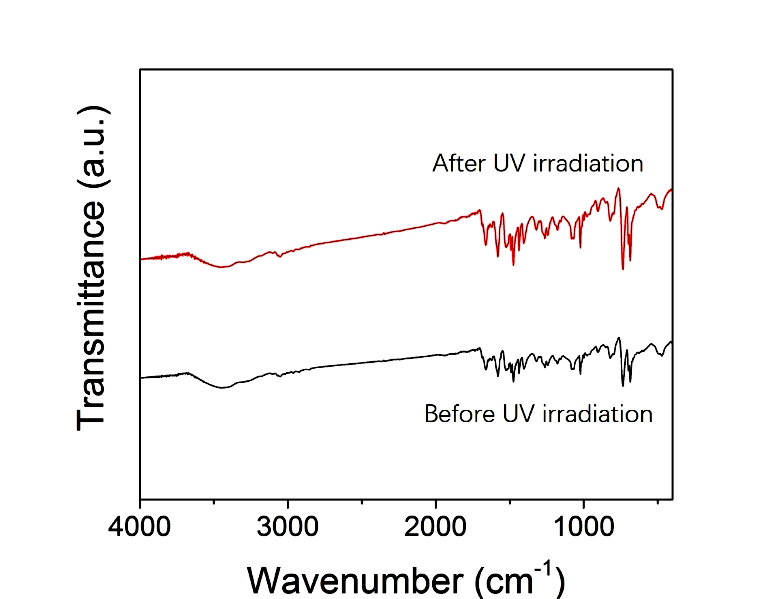


Figure S5. FTIR spectra of **H** before and after UV irradiation for 3 min.


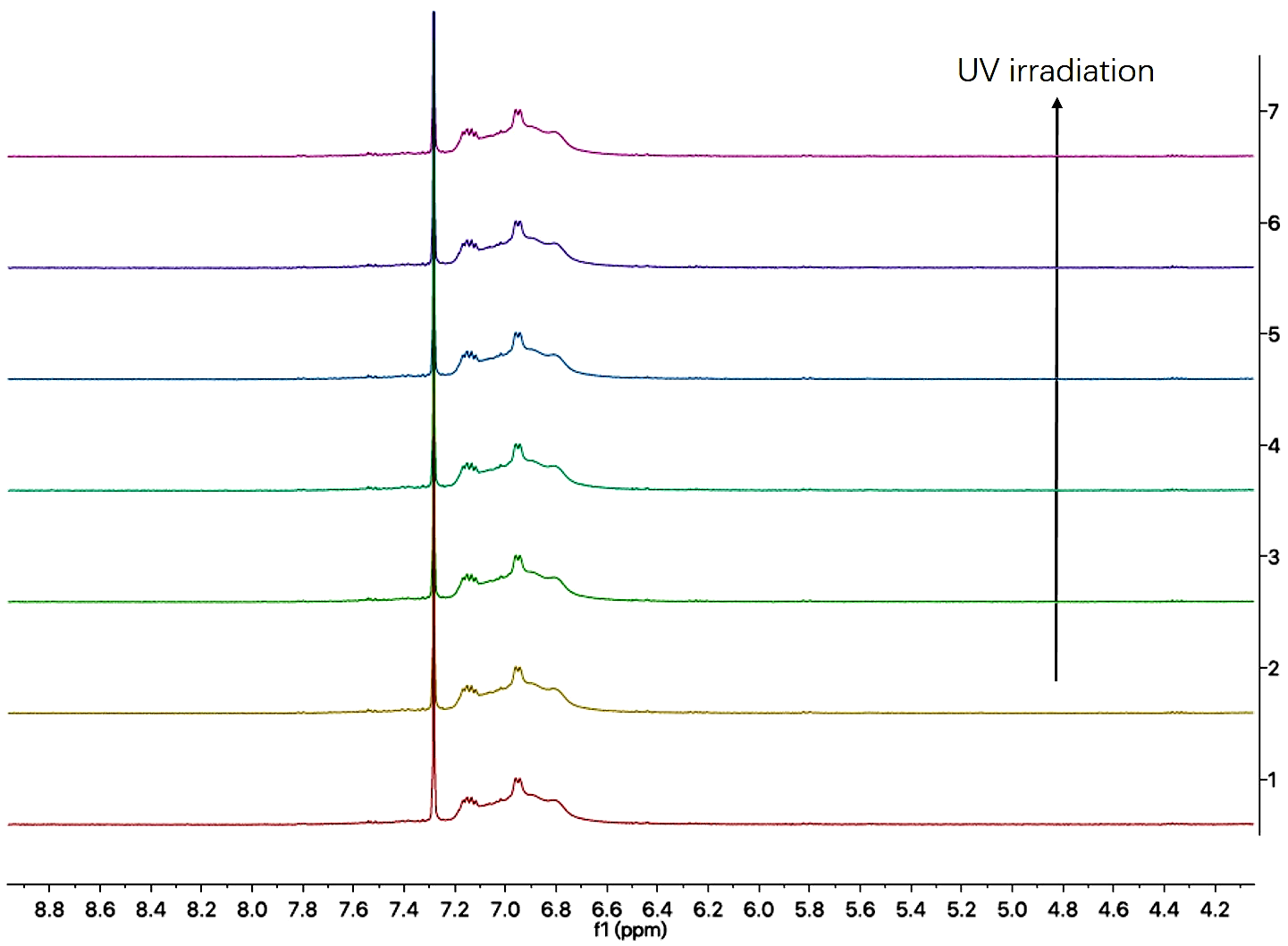


Figure S6. ^1^HNMR spectra of **PH** in CDCl_3_ upon a continuous UV irradiation.


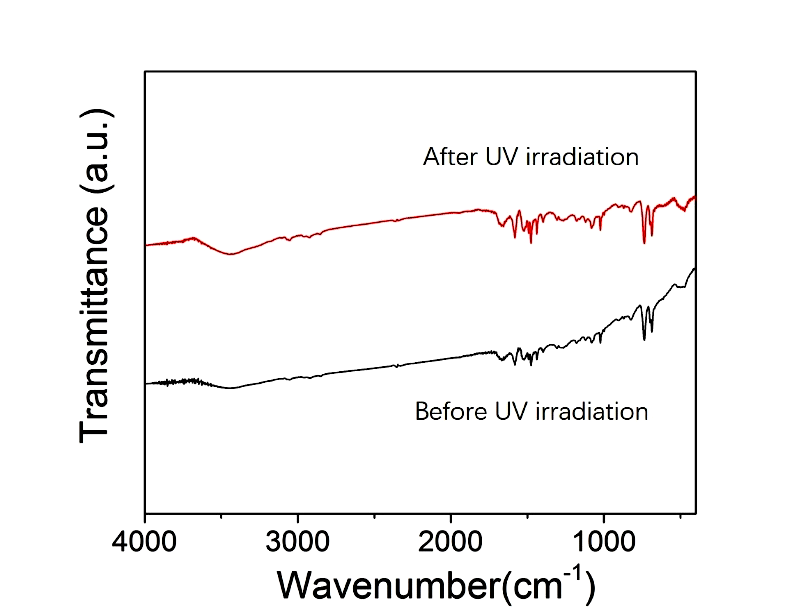


Figure S7. FTIR spectra of **PH** before and after UV irradiation for 3 min.


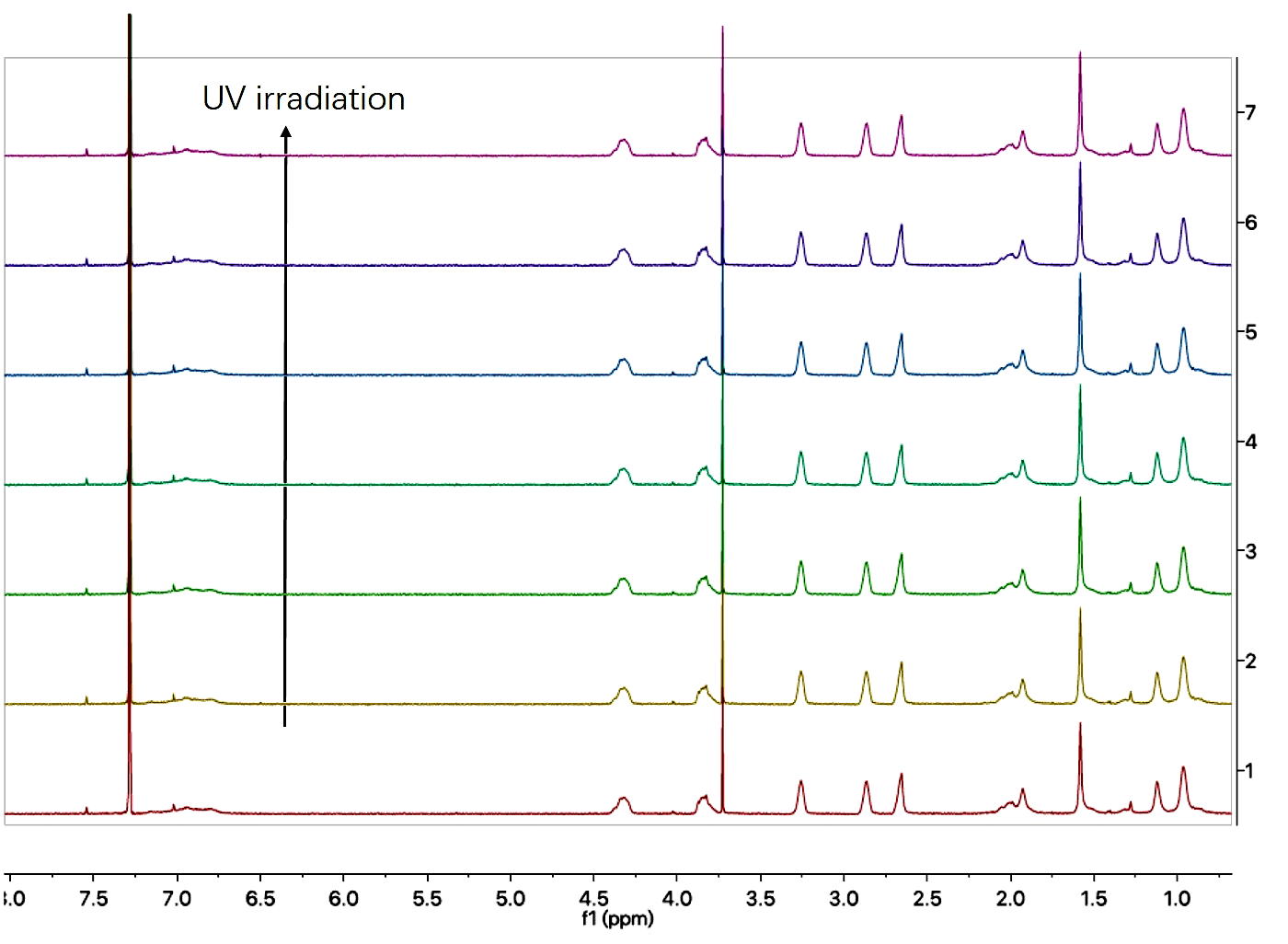


Figure S8. ^1^HNMR spectra of **PH-*b*-PG** in CDCl_3_ upon a continuous UV irradiation.


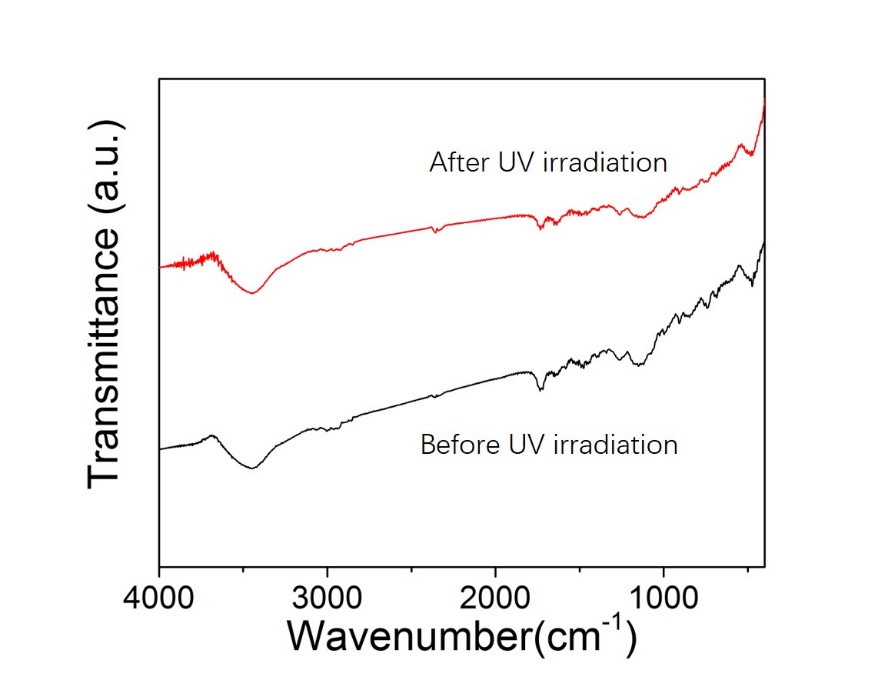


Figure S9. FTIR spectra of **PH-*b*-PG** before and after UV irradiation for 3 min.


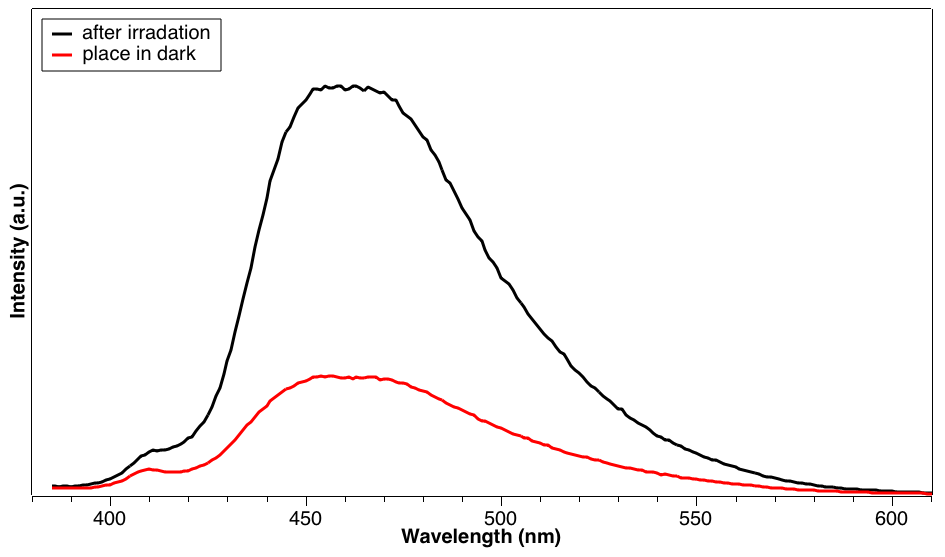


**H**


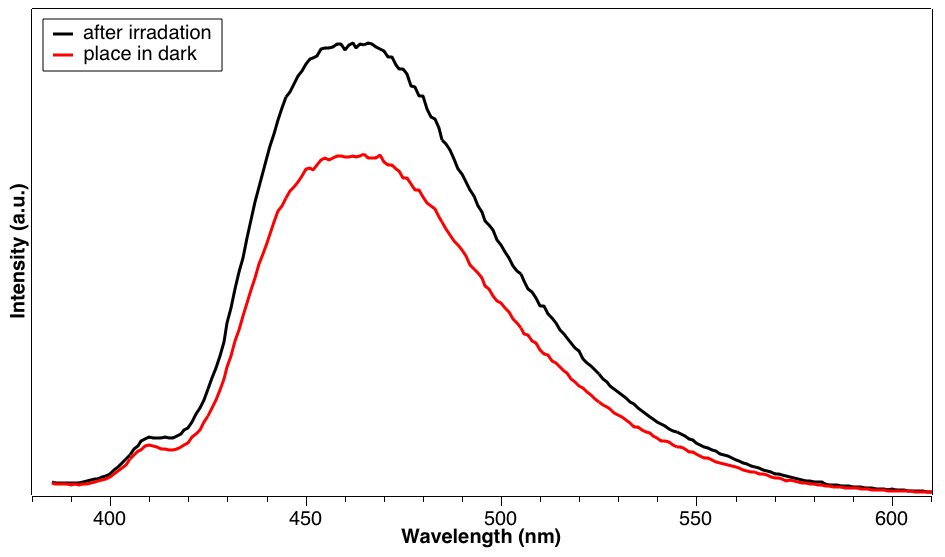


**PH**


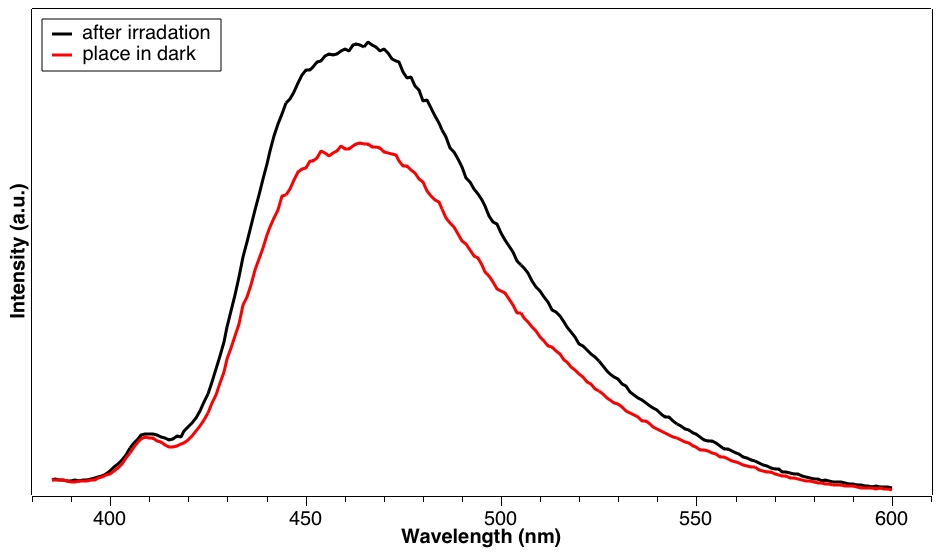


**PH-*b*-PG**

Figure S10. The emission changes of **H**, **PH**, and **PH-*b*-PG** in 1, 4-dioxane after irradiation for 90 s, followed by the relaxation without irradiation for the same period.

Table. S1. Selected torsion angles (θ, deg.) for the different conformers of **H**.

| Molecule | θ[A] | θ[B] | θ[C] | θ[D] | θ[E] | θ [F] |
| --- | --- | --- | --- | --- | --- | --- |
| 1-opt (S_0_) | -119.09 | 118.02 | -117.59 | 117.51 | -118.50 | 118.29 |
| 1-opt (S_1_) | -81.8 | 85.60 | -106.20 | 78.33 | -91.78 | 102.94 |
| 1-opt(T_1_^-^Globle) | -78.76 | 89.90 | -105.23 | 79.14 | -91.66 | 104.47 |
| 1-opt(S_0_-Local) | -119.27 | 118.00 | -117.55 | 117.44 | -118.46 | 118.33 |


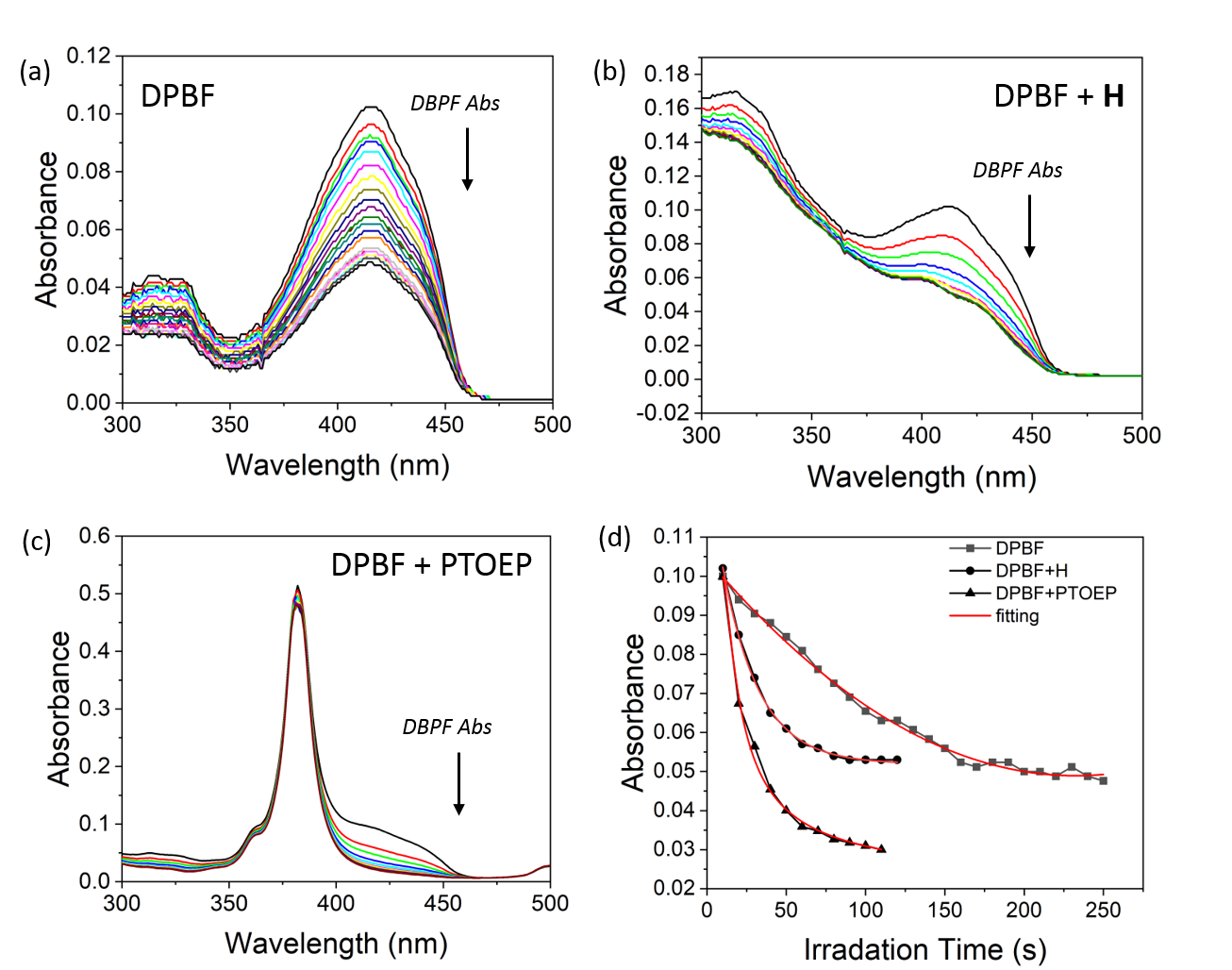


Figure S11. UV-vis absorption spectra after irradiation at 365 nm for different times of (a) DPBF (10 μM), (b) DPBF with H (10 μM), (c) DPBF with PTOEP (10 μM), and the corresponding absorption intensity of DPBF at 410 nm versus the UV irradiation time.

INCLUDEPICTURE "../../../../../../Users/zhu/AppData/Roaming/Tencent/Users/77426127/QQ/WinTemp/RichOle/%5bKI1%7d~J%7bOT(ZXM9$CIDDMOM.png" \* MERGEFORMAT INCLUDEPICTURE "../../../../../../Users/zhu/AppData/Roaming/Tencent/Users/77426127/QQ/WinTemp/RichOle/%5bKI1%7d~J%7bOT(ZXM9$CIDDMOM.png" \* MERGEFORMAT
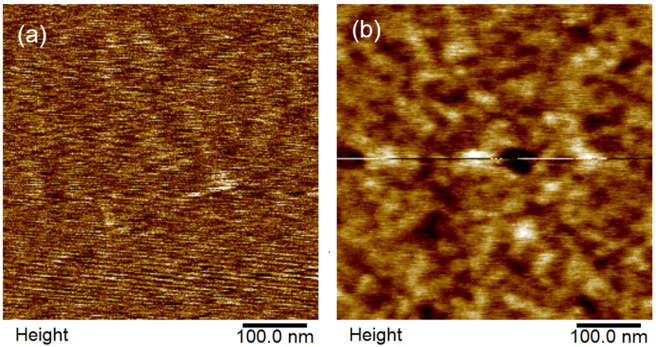


Figure S12. AFM height images of (a) **H** and (b) **PH**.


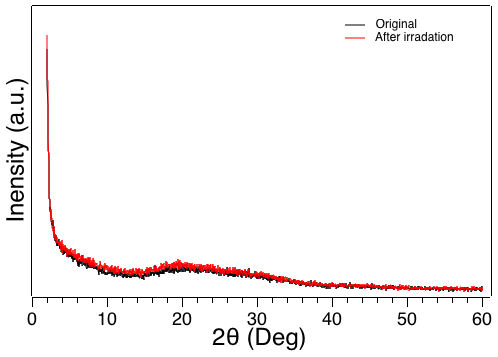


Figure S13. The XRD spectra of **PH-*b*-PG** before and after UV irradiation for 10 min.


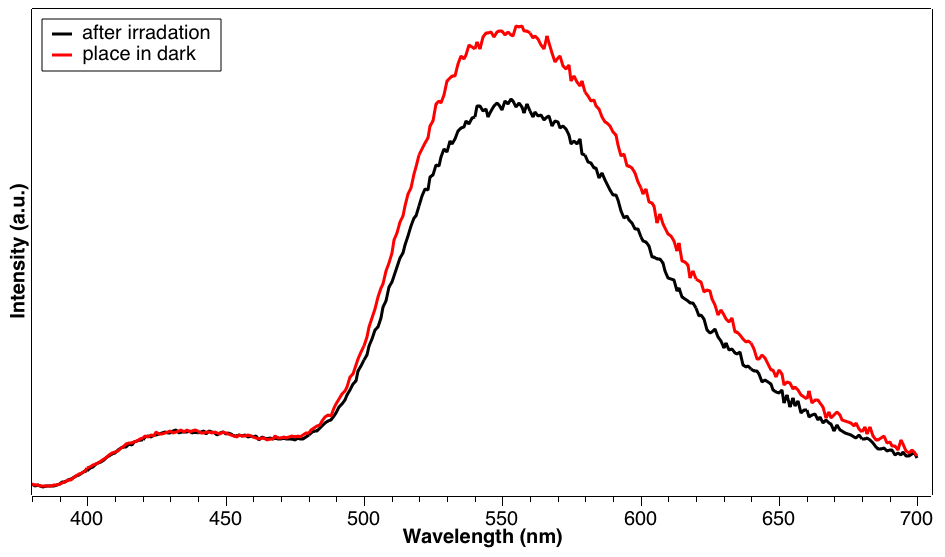


**H**


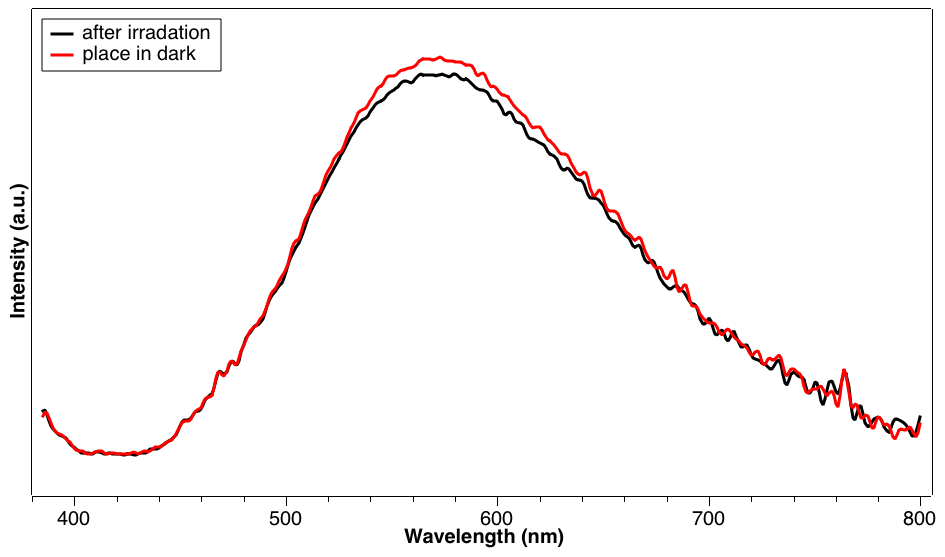


**PH**


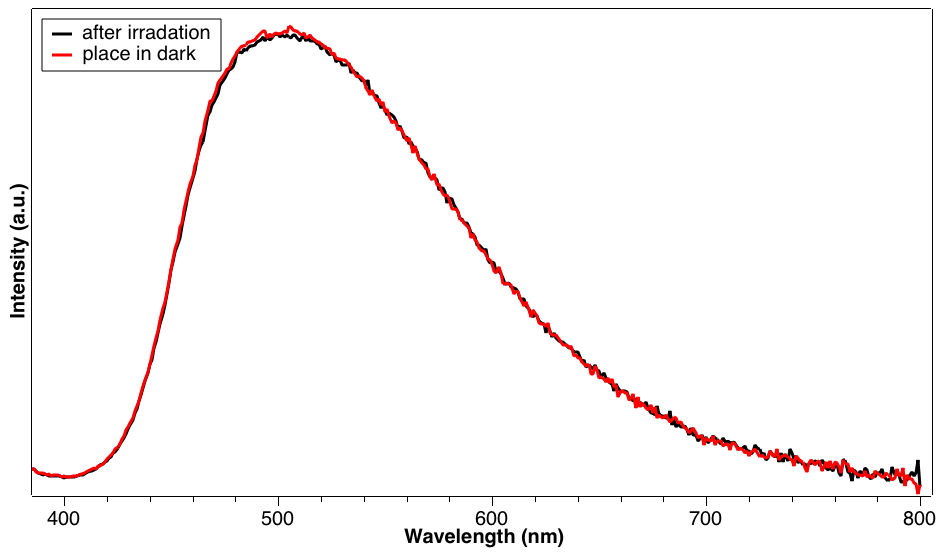


**PH-*b*-PG**

Figure S14. The emission changes of **H**, **PH**, and **PH-*b*-PG** in film state after irradiation for 90 s, followed by the relaxation without irradiation for the same period.

Figure S15. TGA of (a) **H** and (b) **PH-*b*-PG** before and after irradiation, and (c) a partially magnified spectrum of (b).

**References**

[S1] Becke, A. D. Density-functional thermochemistry. III. The role of exact exchange. *J. Chem. Phys.* **98**, 5648-5652 (1993).

[S2] Lee, C., Yang, W. & Parr, R. G. Development of the Colic-Salvetti correlation-energy formula into a functional of the electron density. *Phys. Rev.* *B*, **37**, 785-789 (1998).

[S3] Frisch, M. J., Pople, J. A. & Binkley, J. S. Self-consistent molecular orbital methods 25. Supplementary functions for Gaussian basis Sets. *J. Chem. Phys*. **80**, 3265-3269 (1984).

[S4] Furche, F. & Ahlrichs, R. Adiabatic time-dependent density functional methods for excited state properties. *J. Chem. Phys.* **117**, 7433-7447 (2002).

Appendix


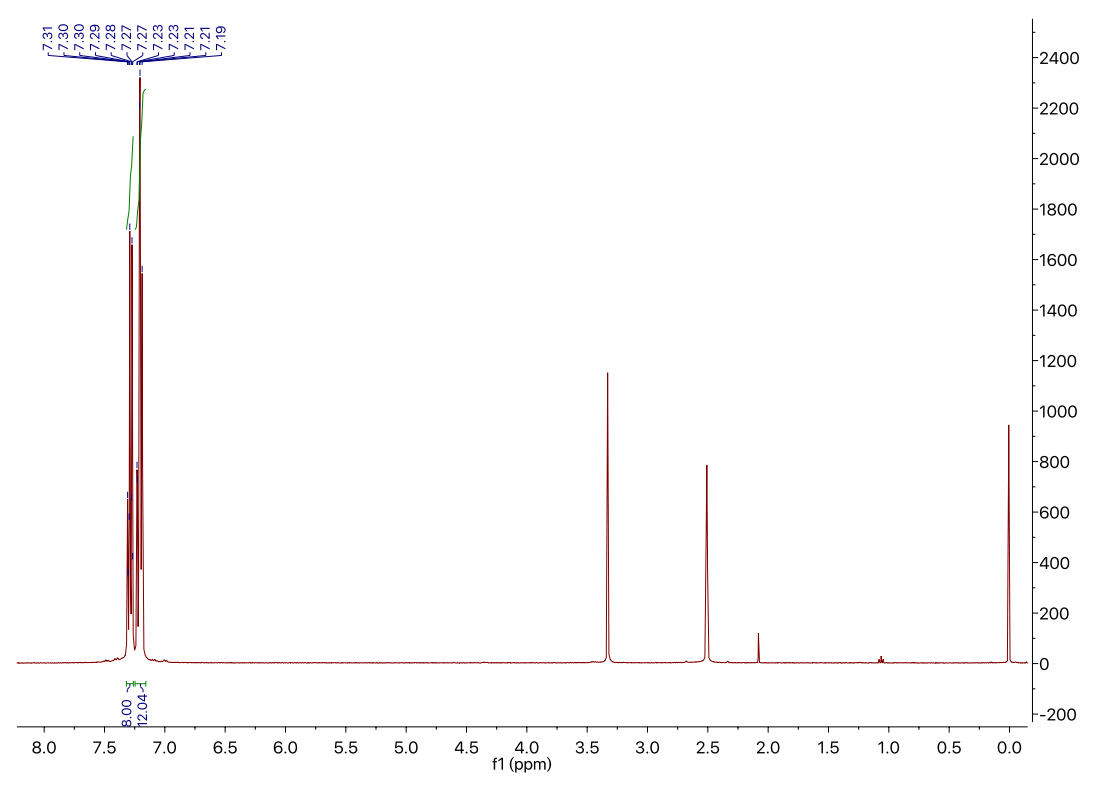


^1^H NMR spectrum (400 MHz, DMSO-d, 298 K) of **1**


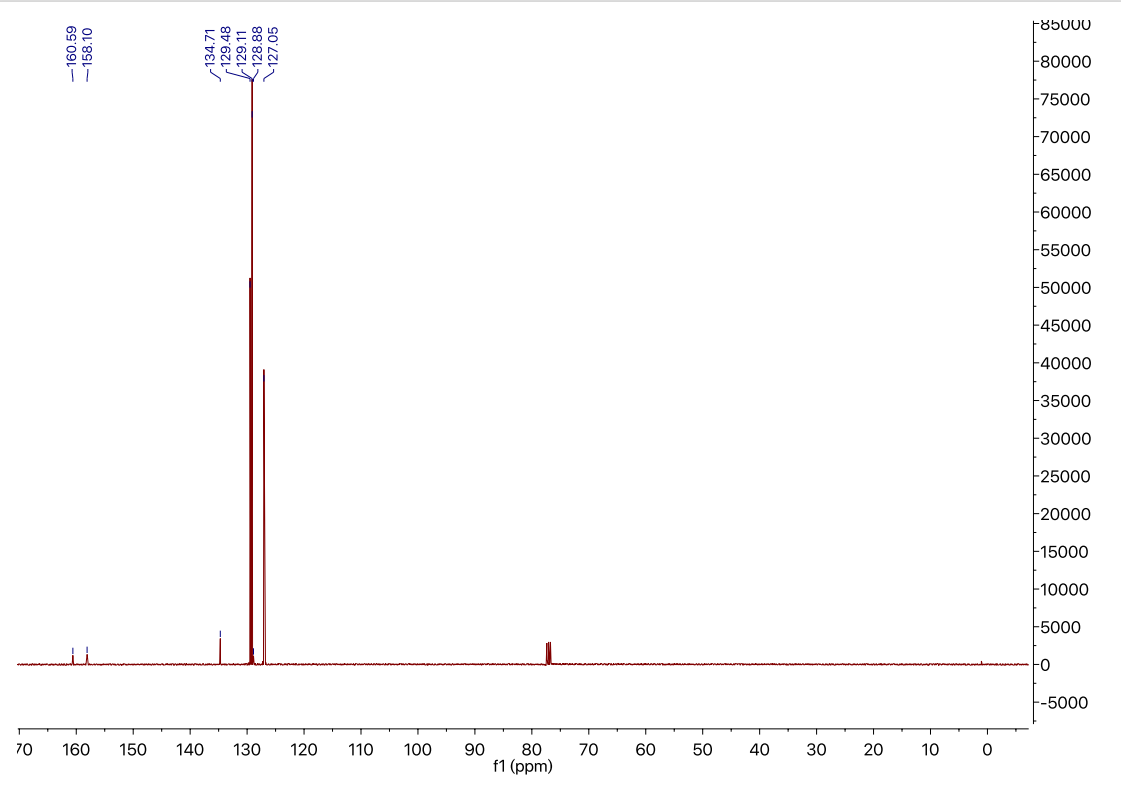


^13^C NMR spectrum (100 MHz, CDCl_3_-d, 298 K) of **1**


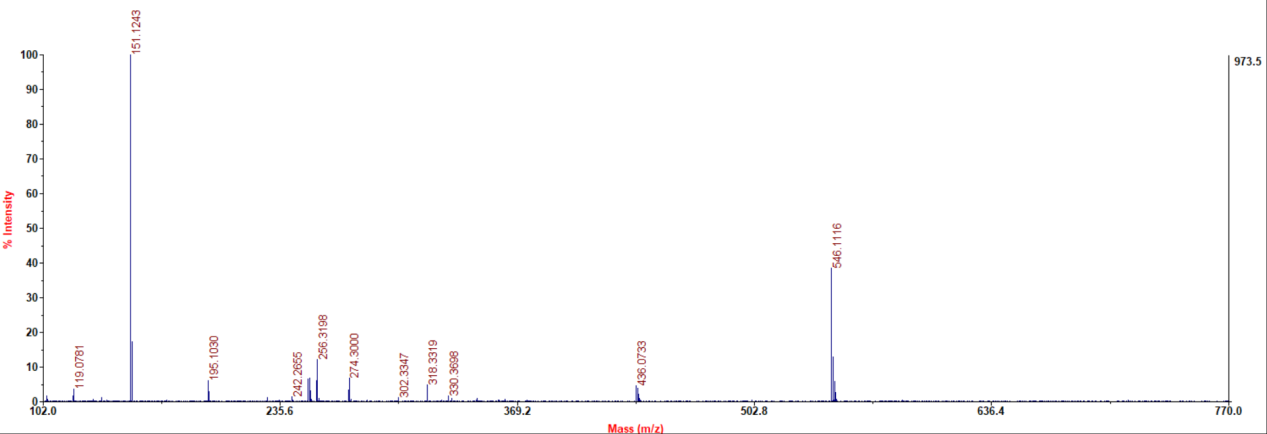


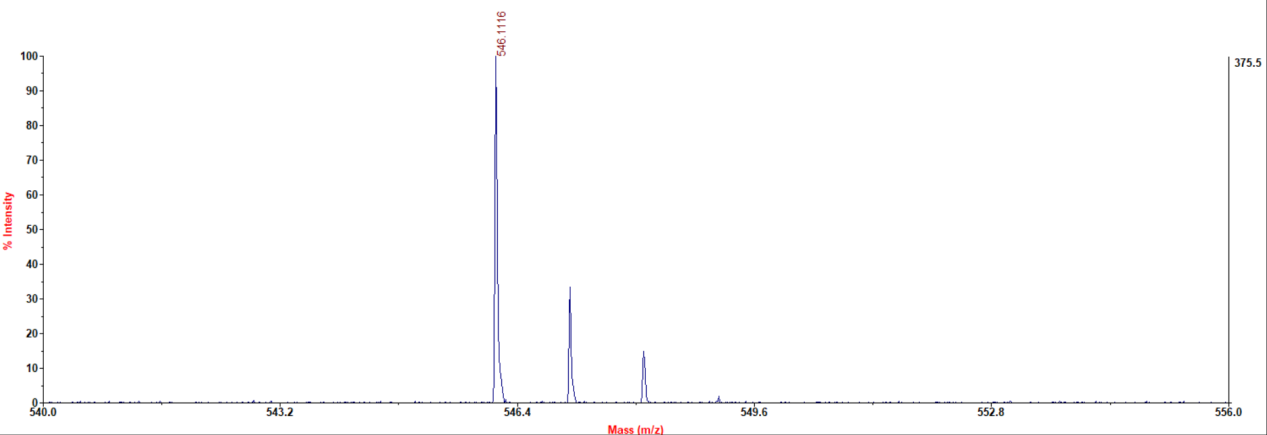


MALDI-TOF MS spectra of **1**


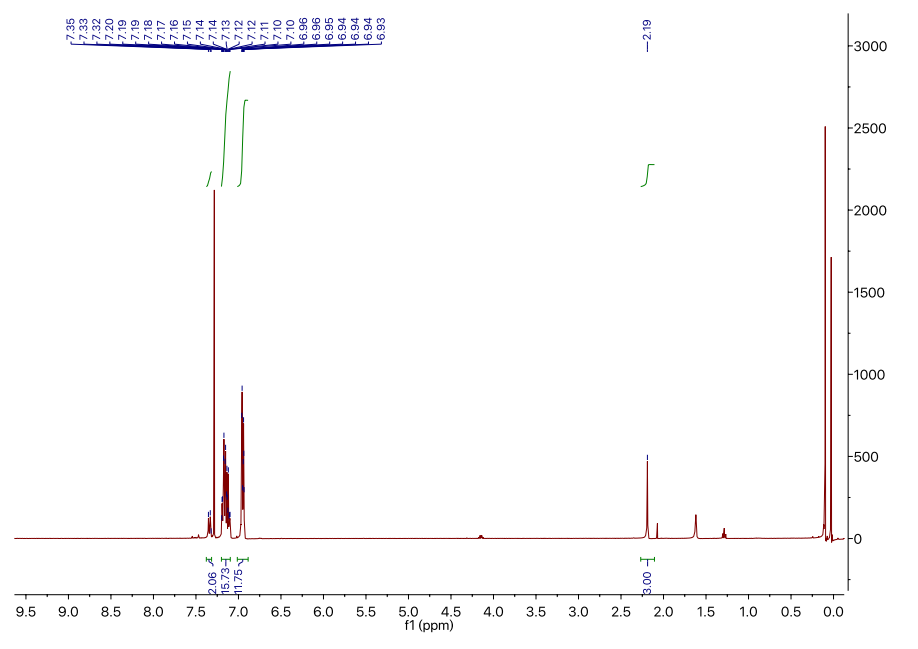


^1^H NMR spectrum (400 MHz, CDCl_3_-d, 298 K) of **2**


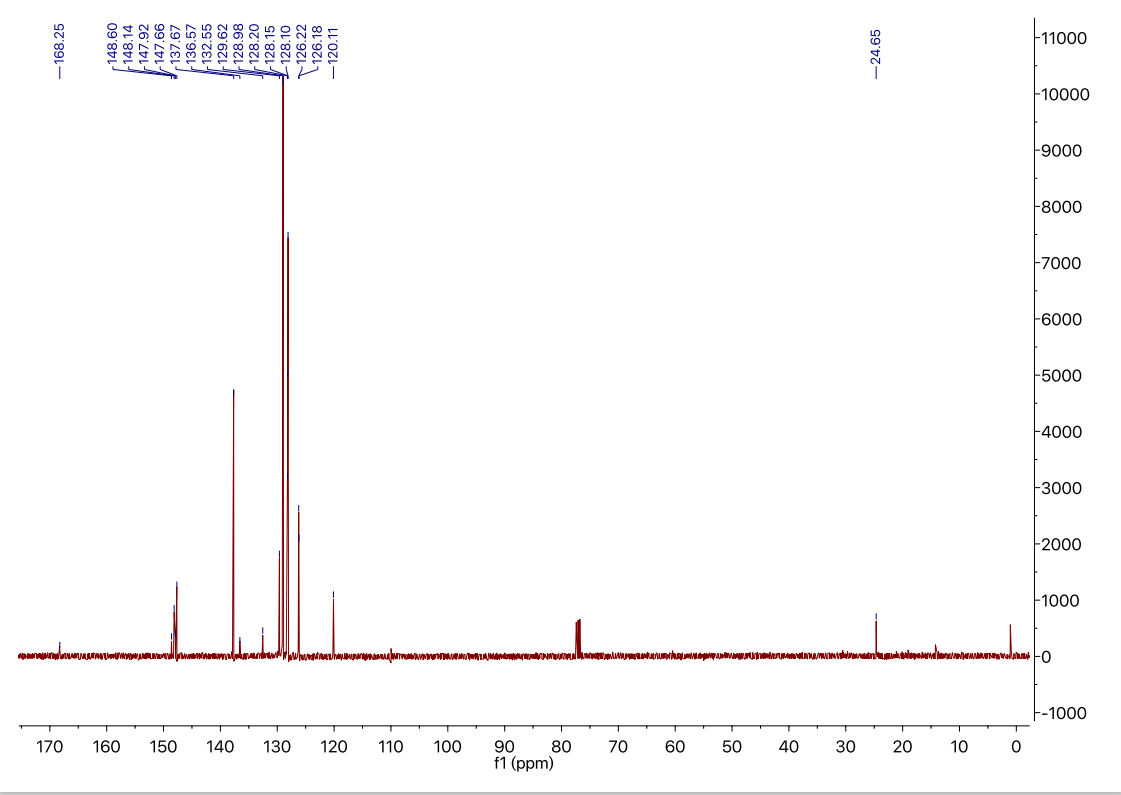


^13^C NMR spectrum (100 MHz, CDCl_3_-d, 298 K) of **2**


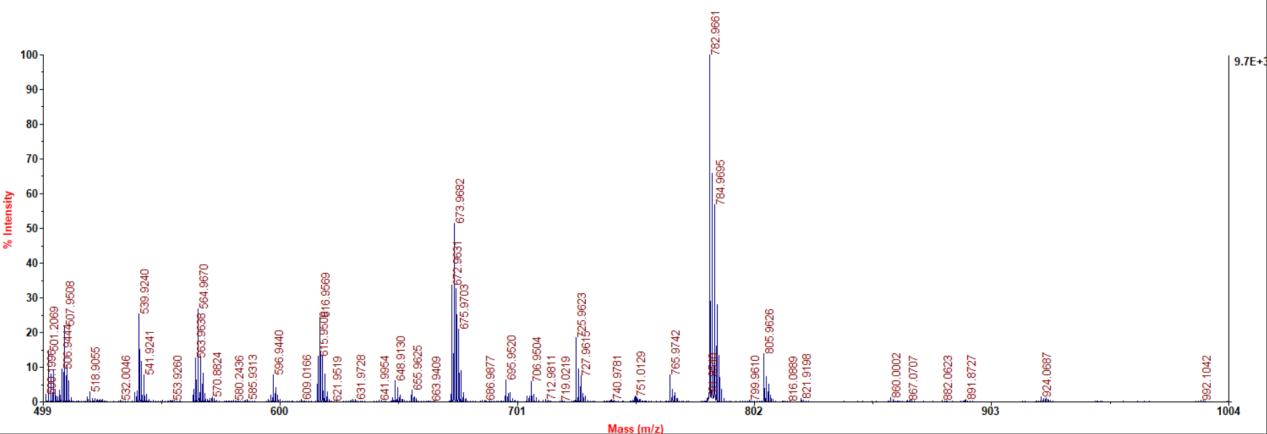


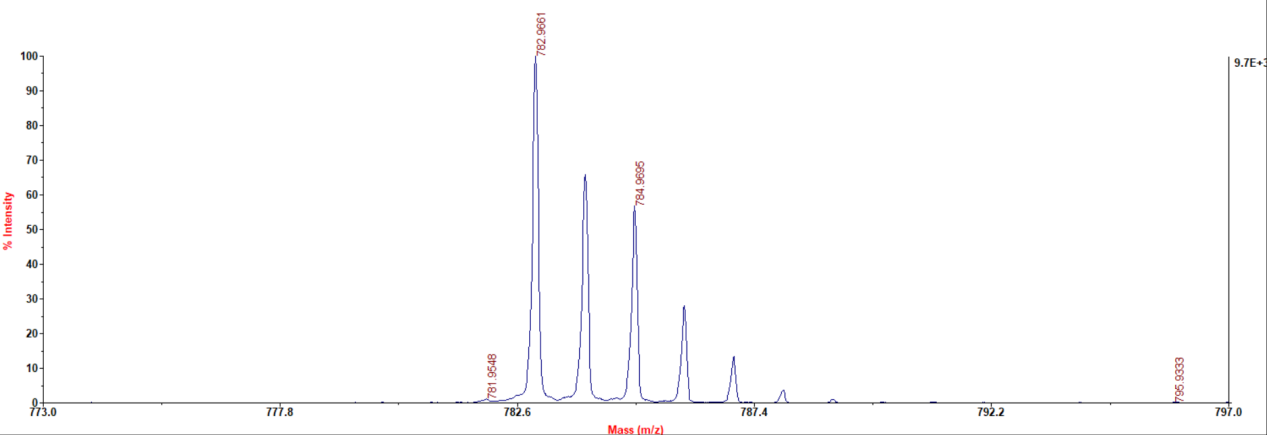


MALDI-TOF MS spectra of **2**


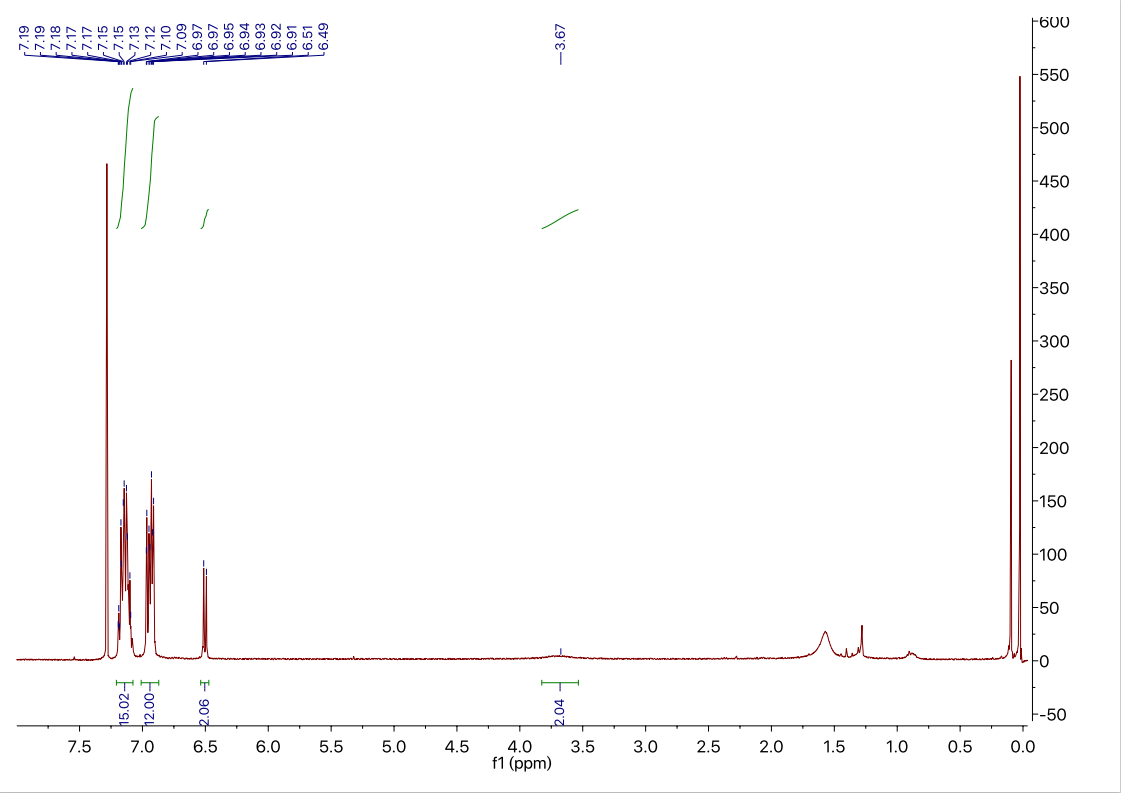


^1^H NMR spectrum (400 MHz, CDCl_3_-d, 298 K) of **3**


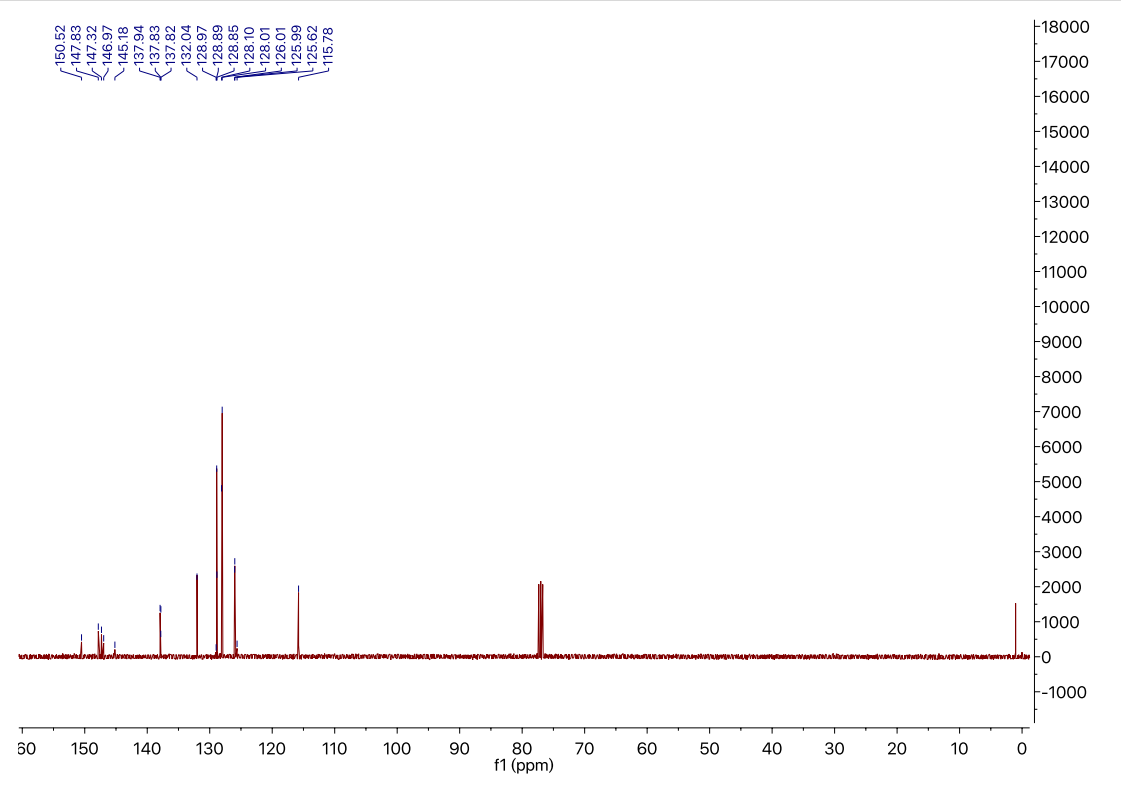


^13^C NMR spectrum (100 MHz, CDCl_3_-d, 298 K) of **3**


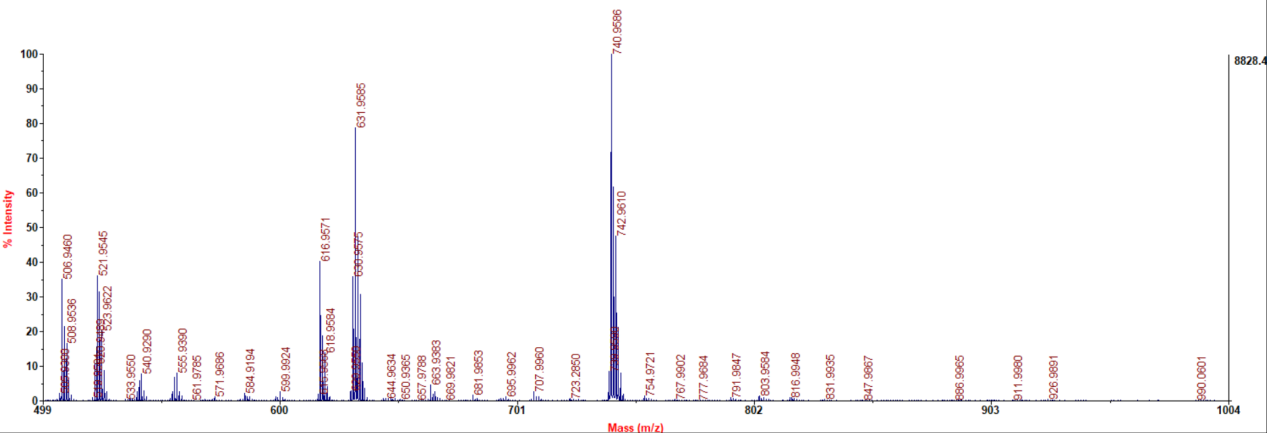


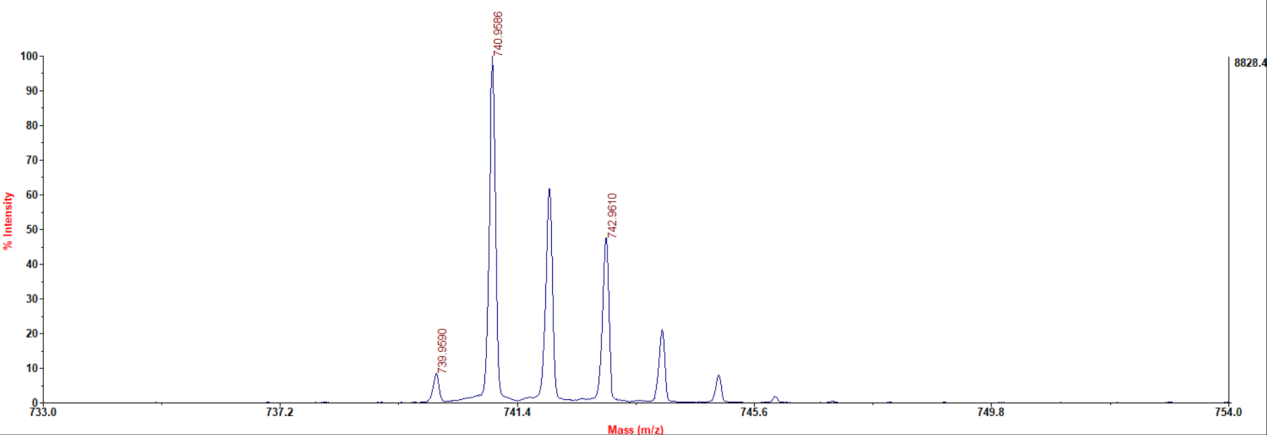


MALDI-TOF MS spectra of **3**


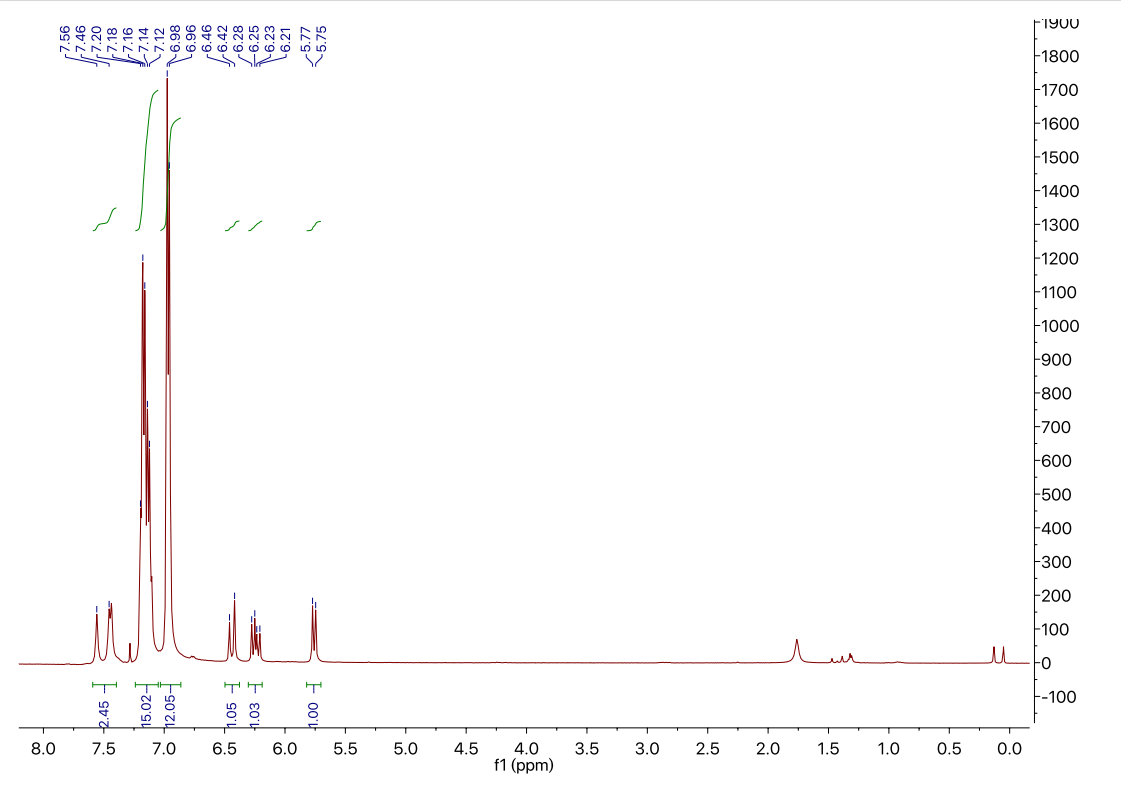


^1^H NMR spectrum (400 MHz, CDCl_3_-d, 298 K) of **H**


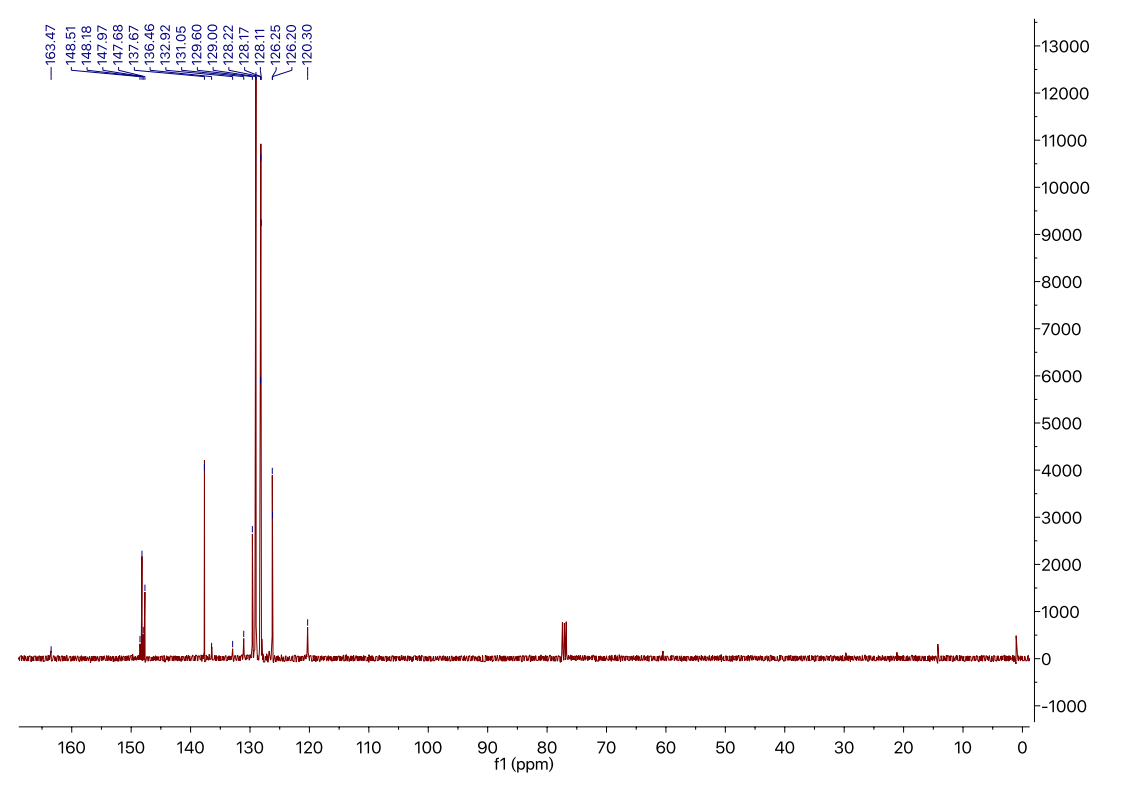


^13^C NMR spectrum (100 MHz, CDCl_3_-d, 298 K) of **H**


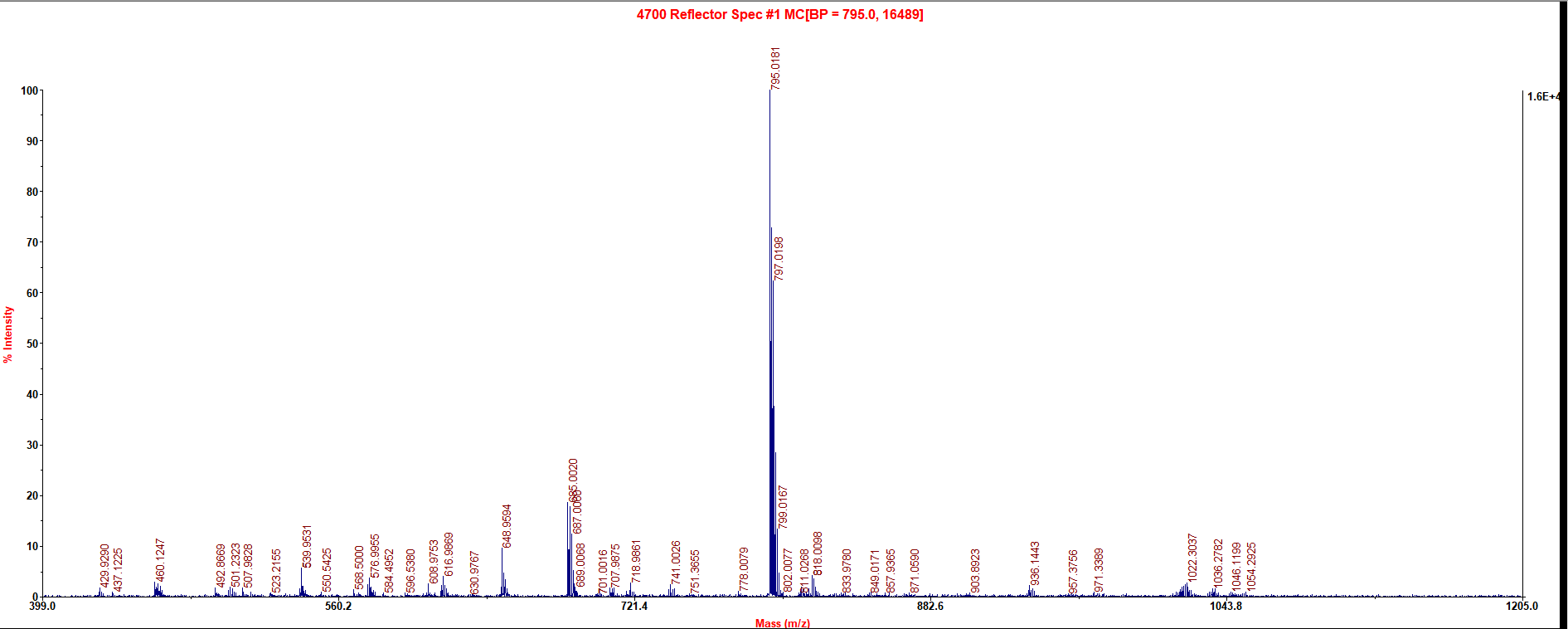


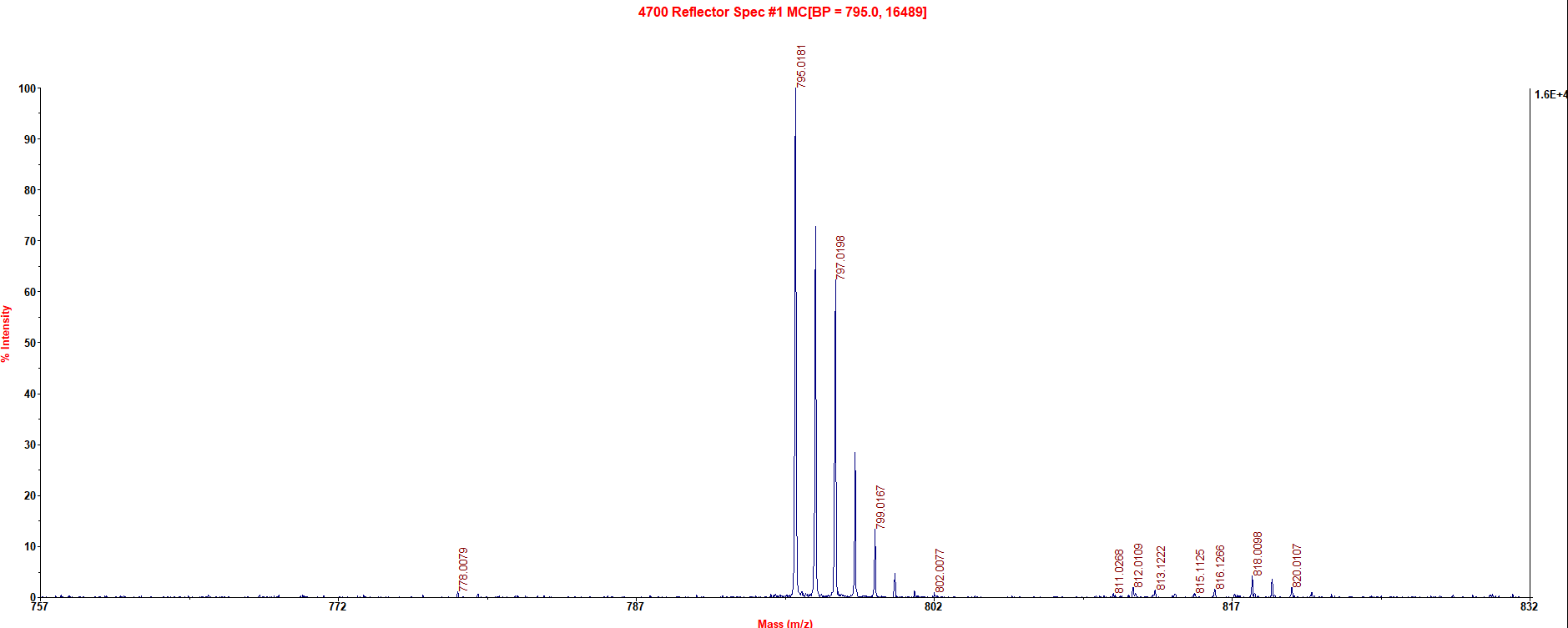


MALDI-TOF MS spectra of **H**


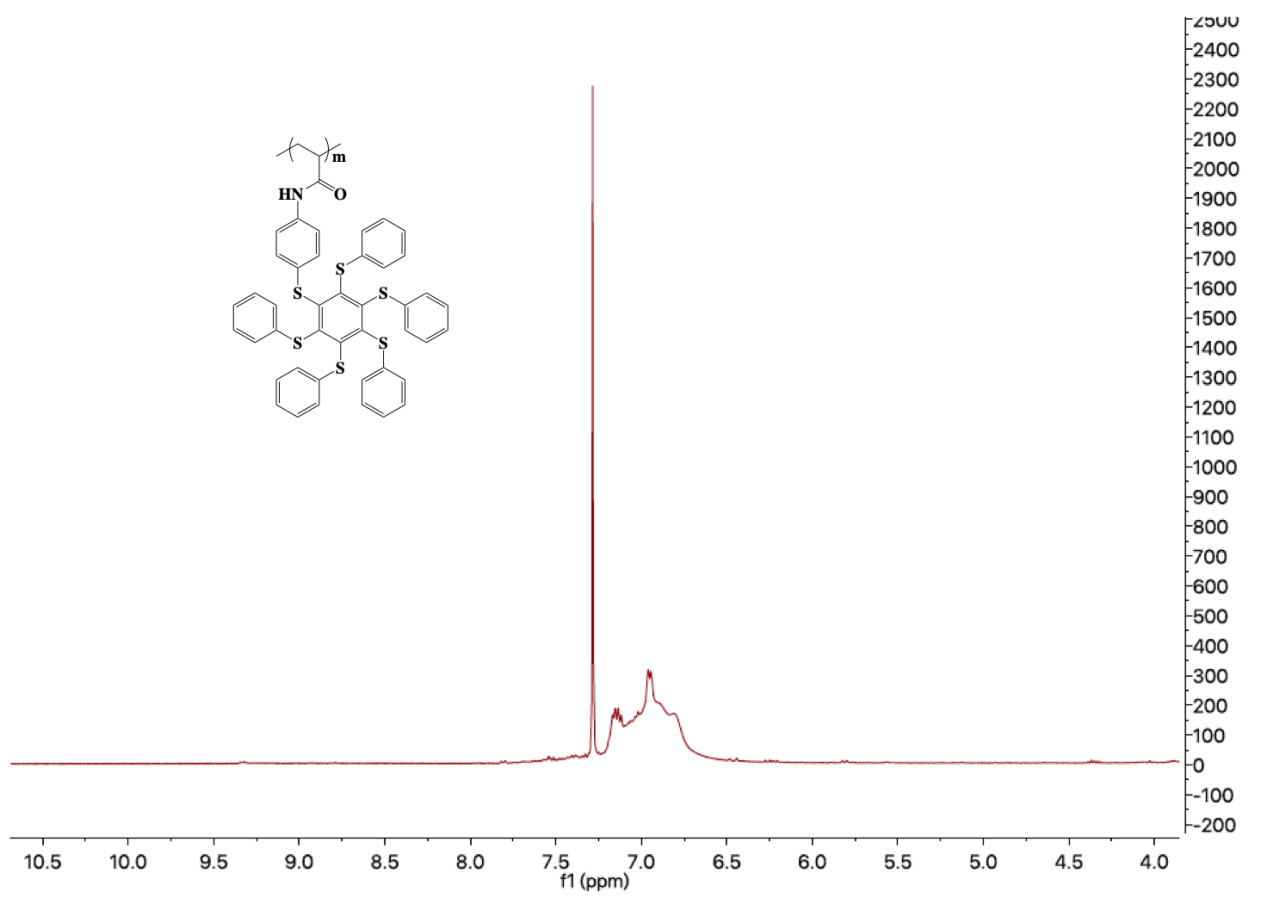


^1^H NMR spectrum (400 MHz, CDCl_3_-d, 298 K) of **PH**


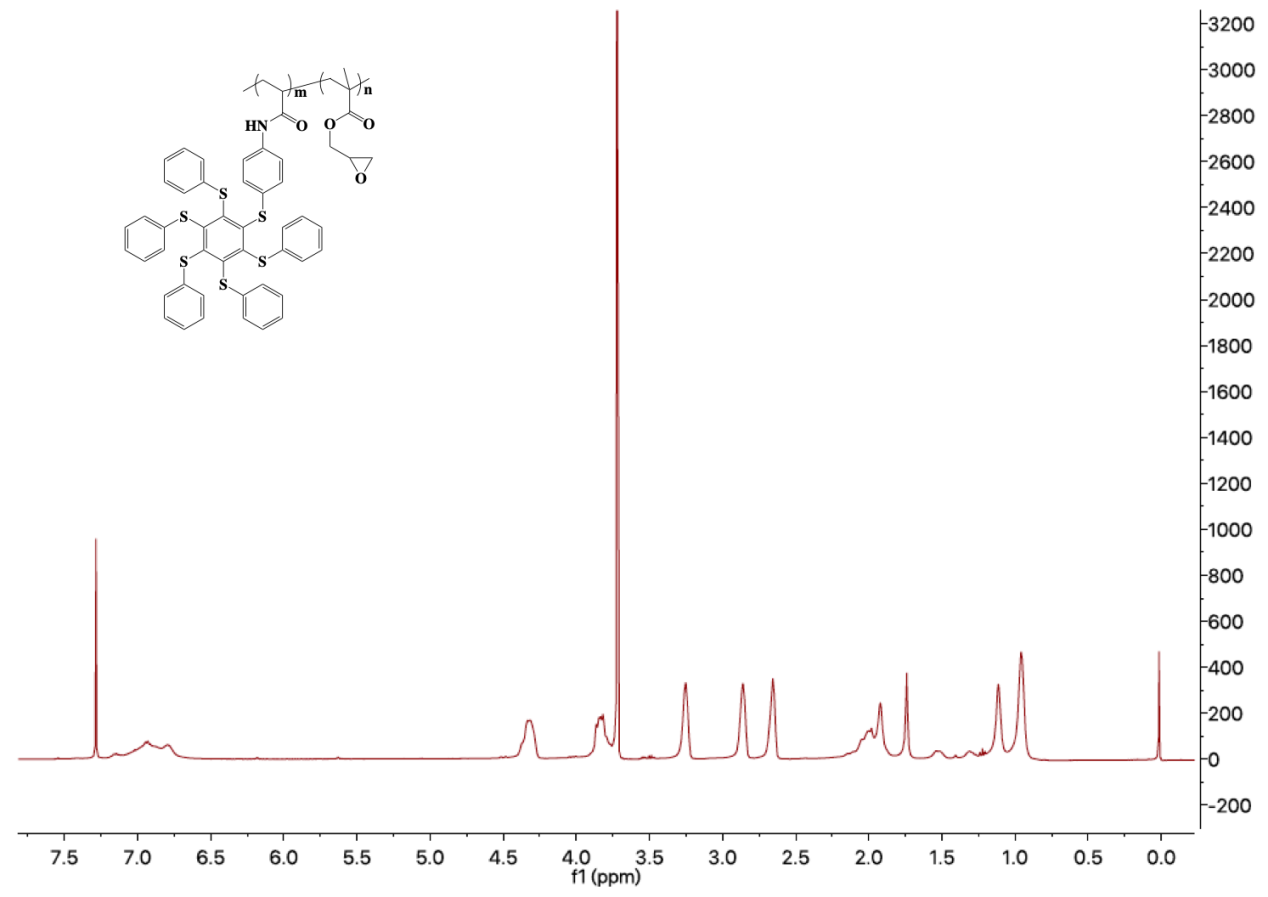


^1^H NMR spectrum (400 MHz, CDCl_3_-d, 298 K) of **PH-b-PG**
